# Supplementary material for: A pooled analysis of the incidence and mortality risk of atrial fibrillation in patients with COVID-19
Source: PeerJ. 2024 Oct 16;12:e18330. doi: 10.7717/peerj.18330 (PMC11490229; doi:10.7717/peerj.18330)
Supplement: Supplemental Information 2 [file peerj-12-18330-s002.docx]

SUPPLEMENTAL FILE

Title: An Pooled Analysis of the incidence and mortality risk of AF in patients with COVID-19

Contents

**Tables**

[Table S1. Search strategy to identify studies reporting the prevalence of AF in patients with COVID-19 and the associated outcomes 3](#_Toc42611083)

[Table S2. Quality assessment scale 5](#_Toc42611084)

[Table S3. Excluded studies with reasons 7](#_Toc42611084)

[Table S4. Detailed demographics and clinical characteristics of the included studies 9](#_Toc42611086)

[Table S5. Quality scores of the included studies 1](#_Toc42611087)2

[Table S6. Sensitivity analysis of pre-existing and new-onset AF rate 1](#_Toc42611088)5

[Table S7. Sensitivity analysis of pre-existing and new-onset AF rate by regions 1](#_Toc42611089)7

[Table S8. Sensitivity analysis of effect of pre-existing and new-onset AF on mortality. 1](#_Toc42611090)9

[Table S9. Meta-regression of AF prevalence in COVID-19 2](#_Toc42611091)0

[Table S10. Meta-regression of effect of pre-existing and new-onset AF on mortality. 21](#_Toc42611092)

[Table S11. Detailed characteristics of the included studies. 22](#_Toc42611092)

**Figures**

[Figure S1. Pooled prevalence of pre-existing rate of AF in COVID-19 25](#_Toc42611097)

[Figure S2. Pooled prevalence of pre-existing rate of AF in mean age≥ 65 years 26](#_Toc42611098)

[Figure S3. Pooled prevalence of pre-existing rate of AF in mean age < 65 years 2](#_Toc42611099)7

[Figure S4. Pooled prevalence of pre-existing rate of AF in COVID-19 in Europe 2](#_Toc42611097)8

[Figure S5. Pooled prevalence of pre-existing rate of AF in COVID-19 in North America 2](#_Toc42611098)9

[Figure S6. Pooled prevalence of pre-existing rate of AF in COVID-19 in Asia 3](#_Toc42611099)0

[Figure S7. Pooled prevalence of pre-existing rate of AF in severe patients 3](#_Toc42611097)1

[Figure S8. Pooled prevalence of pre-existing rate of AF in non-severe patients](#_Toc42611098) 32

[Figure S9. Pooled prevalence of pre-existing rate of AF in sample size < 500 3](#_Toc42611096)3

[Figure S10. Pooled prevalence of pre-existing rate of AF in sample size > 500 34](#_Toc42611097)

[Figure S11. Pooled prevalence of new-onset rate of AF in severe and non-severe patients 35](#_Toc42611098)

[Figure S12. Pooled prevalence of pre-existing AF on all-cause mortality 36](#_Toc42611099)

[Figure S13. Pooled prevalence of pre-existing AF on all-cause mortality by region 3](#_Toc42611099)7

[Figure S14. Pooled prevalence of pre-existing AF on all-cause mortality by study type 38](#_Toc42611099)

[Figure S15. Pooled prevalence of pre-existing AF on all-cause mortality by sample size 39](#_Toc42611099)

[Figure S16. Pooled prevalence of pre-existing AF on all-cause mortality by age 4](#_Toc42611099)0

[Figure S17. Pooled prevalence of pre-existing AF on all-cause mortality by disease level 4](#_Toc42611099)1

[Figure S18. Pooled prevalence of new-onset AF on all-cause mortality 4](#_Toc42611099)2

[Figure S19. Publication bias of studies on the AF prevalence in COVID-19 patients 4](#_Toc42611099)3

[Figure S20. Publication bias of studies on the effect of AF on mortality in COVID-19 patients 44](#_Toc42611099)

**[References](#_Toc42611100)** [45](#_Toc42611100)

# Table S1. Search strategy to identify studies reporting the prevalence of AF in patients with COVID-19 and the associated outcomes.

| **Literature databases** | **Search items** | **Items found** |
| --- | --- | --- |
| MEDLINE via Pubmed | #1  “atrial fibrillation”[MeSH Terms] OR “atrial fibrillation”[Title/Abstract] OR “auricular fibrillation”[Title/Abstract] OR “AF”[Title/Abstract] OR “AFib”[Title/Abstract]  #2  “COVID-19”[MeSH Terms] OR “COVID-19” [Title/Abstract] OR “SARS-CoV-2” [Title/Abstract] OR “coronavirus disease 2019” [Title/Abstract] OR “Severe acute respiratory syndrome coronavirus 2”[Title/Abstract] OR “2019-nCoV”[Title/Abstract]  #1 AND #2 | 1439 |
| EMBASE | #1  ‘atrial fibrillation’/exp OR ‘atrial fibrillation’:ti,ab,kw OR ‘auricular fibrillation’: ti,ab,kw OR ‘AF’/exp OR ‘AFib’: ti,ab,kw  #2  ‘COVID-19’:ti,ab,kw OR ‘SARS-CoV-2’:ti,ab,kw OR ‘coronavirus disease 2019’:ti,ab,kw OR ‘Severe acute respiratory syndrome coronavirus 2’:ti,ab,kw OR ‘2019-nCoV’:ti,ab,kw  #1 AND #2 | 261 |
| COCHRANE | #1  MeSH descriptor: [atrial fibrillation] OR atrial fibrillation: ti,ab,kw OR auricular fibrillation: ti,ab,kw OR AF: ti,ab,kw OR AFib: ti,ab,kw  #2  COVID-19:ti,ab,kw OR SARS-CoV-2:ti,ab,kw OR coronavirus disease 2019:ti,ab,kw OR Severe acute respiratory syndrome coronavirus 2:ti,ab,kw OR 2019-nCoV:ti,ab,kw  #1 AND #2 | 125 |
| Overall |  | 1825 |
| Duplication |  | 89 |

**Table S2. Quality assessment scale**

| **Bias type** | **Selection**  **(sample population)** | **Selection**  **(sample size)** | **Selection (participation rate)** | **Performance bias (outcome assessment)** | **Performance bias (analytical methods to control for bias)** |
| --- | --- | --- | --- | --- | --- |
| **Low risk**  **(score=2)** | 1) Sample from the general population, not a select group;  2) Consecutive unselected population;  3) Rationale for case and control selection explained. | 1) Sample size calculation performed and adequate. | 1) High response rate (>85%). | 1) Diagnosis using consistent criteria and direct examination. | 1) Analysis appropriate for the type of sample (subgroup analysis/regression etc.) |
| **Moderate risk (score=1)** | 1) Sample selected from large population but selection criteria not defined;  2) Sample selection ambiguous but may be representative;  3) Rationale for cases and controls not explained;  4) Eligibility criteria not explained;  5) Analysis to adjust for sampling strategy bias. | 1) Sample size calculation performed and reasons for not meeting sample size given;  2) Sample size calculation not performed but all eligible persons studied. | 1) Moderate response rate (70-85%). | 1) Assessment from administrative database or register;  2) Assessment from hospital record or interviewer. | 1) Analysis does not account for common adjustment. |
| **High risk (score=0)** | 1) Highly select population making it difficult to generalise finding;  2) Sample selection ambiguous and sample unlikely to be representative. | 1) Sample size estimation unclear or only sub-sample studied. | 1) Low response rate (<70%);  2) Response rate not reported. | 1) Assessment from non-validated data or generic estimate from the overall population. | 1) Data confusing. |

#

# Table S3. Excluded studies with reasons

| **Excluded Studies** | **Reason for exclusion** |
| --- | --- |
| Yenercag, et al. 2021^[1](#_ENREF_1)^ | No prevalence data |
| Wenzler, et al. 2020^[2](#_ENREF_2)^ | Small sample size |
| Wang, et al. 2021^[3](#_ENREF_3)^ | No prevalence data |
| Wang, et al. 2020^[4](#_ENREF_4)^ | No prevalence data |
| Wallentin, et al. 2020^[5](#_ENREF_5)^ | No prevalence data |
| Vila-Corcoles, et al. 2020^[6](#_ENREF_6)^ | No prevalence data |
| Uribarri, et al. 2021^[7](#_ENREF_7)^ | No prevalence data |
| Tiwari, et al. 2020^[8](#_ENREF_8)^ | No prevalence data |
| Szarpak, et al. 2021^[9](#_ENREF_9)^ | Atrial fibrillation/atrial flutter |
| Sotiriou, et al. 2021^[10](#_ENREF_10)^ | No prevalence data |
| Sala, et al. 2020^[11](#_ENREF_11)^ | arrhythmias (No AF) |
| Sabatino, et al. 2020^[12](#_ENREF_12)^ | No prevalence data |
| Rivera-Caravaca, et al. 2021^[13](#_ENREF_13)^ | No prevalence data |
| Peltzer, et al. 2020^[14](#_ENREF_14)^ | No single AF data |
| Patel, et al. 2021^[15](#_ENREF_15)^ | No prevalence data |
| OiShea, et al. 2021^[16](#_ENREF_16)^ | No prevalence data |
| Nanjo, et al. 2020^[17](#_ENREF_17)^ | No prevalence data |
| Musikantow, et al. 2021^[18](#_ENREF_18)^ | Atrial fibrillation/atrial flutter |
| Molina, et al. 2021^[19](#_ENREF_19)^ | No prevalence data |
| Mizuno, et al. 2021^[20](#_ENREF_20)^ | No prevalence data |
| Lip, et al. 2021^[21](#_ENREF_21)^ | No prevalence data |
| Holt, et al. 2020^[22](#_ENREF_22)^ | No prevalence data |
| Hedner, et al. 2020^[23](#_ENREF_23)^ | No prevalence data |
| Genovesi, et al. 2021^[24](#_ENREF_24)^ | No prevalence data |
| Gao, et al. 2021^[25](#_ENREF_25)^ | No prevalence data |
| Fumagalli, et al. 2021^[26](#_ENREF_26)^ | No prevalence data |
| Denas, et al. 2021^[27](#_ENREF_27)^ | No prevalence data |
| Chaudhary, et al. 2021^[28](#_ENREF_28)^ | Atrial fibrillation/atrial flutter |
| Changal, et al. 2021^[29](#_ENREF_29)^ | No prevalence data |
| Butt, et al. 2021^[30](#_ENREF_30)^ | No prevalence data |
| Buckley, et al. 2021^[31](#_ENREF_31)^ | No prevalence data |
| Brojakowska, et al. 2021^[32](#_ENREF_32)^ | No prevalence data |
| Boytsov, et al. 2021^[33](#_ENREF_33)^ | No prevalence data |
| Bhatia, et al. 2021^[34](#_ENREF_34)^ | Atrial fibrillation/atrial flutter |
| Barbhaiya, et al. 2021^[35](#_ENREF_35)^ | No prevalence data |
| Abrams, et al. 2020 ^[36](#_ENREF_36)^ | No prevalence data |

#

# Table S4. Detailed demographics and clinical characteristics of the included studies

| **Study** | **Mean**  **age (y)** | **Female (%)** | **HF**  **(%)** | **HBP (%)** | **DM (%)** | **TIA (%)** | **CVD (%)** | **BMI (kg/m^2^)** | **Co-antiplatelet agents** | **Beta blocker agents** | **co-ACEI** | **co-ARB** |
| --- | --- | --- | --- | --- | --- | --- | --- | --- | --- | --- | --- | --- |
| Zhou, 2021 | 44.80 | 49.87 | 0.11 | 13.52 | 1.66 | NR | NR | NR | 4.27 | 4.61 | 3.60 | 3.35 |
| Vergara, 2021 | 71.10 | 32.30 | NR | 57.40 | 16.30 | NR | 9.30 | NR | 23.20 | 29.40 | 23.90 | 18.30 |
| Velilla-Alonso, 2021 | 76.00 | 44.60 | NR | 78.60 | 28.60 | 25.00 | NR | NR | NR | NR | NR | NR |
| Turgay Yildirim, 2021 | 49.20 | 38.80 | NR | 28.10 | 15.10 | NR | 7.90 | NR | NR | NR | NR | NR |
| Terlecki, 2021 | 63.00 | 48.80 | 10.30 | 27.40 | 25.70 | NR | 16.80 | 5.04 | 19.90 | 40.70 | 18.90 | 7.20 |
| Sanz, 2021 | 64.90 | 40.50 | 7.50 | 44.60 | 14.90 | NR | NR | NR | NR | NR | NR | NR |
| Saleh, 2021 | 60.70 | 28.00 | NR | 56.80 | 61.00 | NR | NR | NR | 18.00 | 11.40 | NR | NR |
| Qureshi, 2021 | 49.70 | 54.40 | 12.50 | 47.70 | 30.80 | NR | NR | NR | NR | NR | NR | NR |
| Puttegowda, 2021 | 56.62 | NR | 28.40 | 36.20 | 40.50 | NR | NR | NR | NR | NR | NR | NR |
| Phelps, 2021 | 63.50 | 50.00 | 6.60 | 31.30 | 14.60 | NR | NR | NR | 9.80 | 15.70 | 25.20 | NR |
| Paris, 2021 | 67.40 | 30.30 | NR | 57.20 | 23.30 | NR | NR | NR | NR | 38.00 | NR | NR |
| Palmieri, 2020 | NR | NR | NR | 68.30 | 30.10 | NR | NR | NR | NR | NR | NR | NR |
| Pagnano, 2021 | 73.10 | 37.20 | 19.70 | 74.70 | NR | NR | 4.90 | NR | NR | NR | NR | NR |
| Ozdemir, 2021 | 51.00 | 42.30 | 5.20 | 33.50 | 17.40 | NR | NR | NR | 18.70 | 13.90 | 21.90 | NR |
| Omar, 2021 | 58.00 | 48.10 | 4.60 | 34.40 | 11.00 | NR | 3.10 | NR | NR | NR | NR | NR |
| Mesquita, 2021 | 73.50 | 29.70 | 10.90 | 64.10 | 34.30 | NR | 16.00 | NR | NR | NR | NR | NR |
| Mathew, 2021 | 55.66 | 22.59 | NR | 61.29 | 54.83 | NR | NR | NR | NR | NR | NR | NR |
| Lee, 2021 | 71.90 | 46.10 | 36.90 | 75.40 | 35.40 | NR | NR | NR | NR | NR | NR | NR |
| Lazcano, 2021 | 57.10 | 58.50 | 2.92 | 32.40 | 12.50 | NR | NR | NR | NR | NR | NR | NR |
| Lala, 2020 | 66.40 | 40.40 | 10.10 | 8.30 | 26.30 | NR | 16.60 | NR | NR | NR | NR | NR |
| Kuno, 2022 | 63.10 | 43.70 | 5.10 | 31.30 | 21.00 | NR | NR | NR | NR | NR | NR | NR |
| Kragholm, 2021 | 57.00 | NR | 3.70 | 25.20 | 10.20 | NR | NR | NR | NR | NR | NR | NR |
| Koutroumpakis, 2021 | 57.00 | 56.30 | 10.50 | NR | 40.90 | NR | 10.10 | NR | NR | NR | NR | NR |
| Kanthasamy, 2021 | 59.00 | 17.00 | 4.00 | 56.00 | 42.00 | NR | 16.00 | NR | NR | NR | NR | NR |
| Inciardi, 2020 | 67.00 | 19.00 | 21.00 | 64.00 | 31.00 | NR | NR | NR | NR | NR | NR | 19.00 |
| Henein, 2021 | 67.00 | 37.00 | NR | 54.00 | 26.00 | NR | NR | NR | 15.00 | 21.00 | 34.00 | NR |
| Harrison, 2021 | 64.20 | 53.60 | 6.70 | 42.30 | 22.10 | NR | NR | NR | NR | NR | NR | NR |
| Garcia-Granja, 2021 | 68.10 | 44.00 | NR | 50.00 | 18.00 | NR | NR | NR | NR | 18.00 | NR | NR |
| Fumagalli, 2021 | 81.00 | 50.50 | 27.40 | 75.00 | 32.70 | NR | 62.90 | NR | NR | NR | NR | NR |
| Ergun, 2020 | 71.00 | 29.00 | 15.70 | 70.60 | 36.70 | NR | 26.20 | 26.00 | NR | NR | NR | NR |
| Chanseaume, 2021 | 52.46 | 52.23 | 1.52 | NR | NR | NR | NR | NR | NR | NR | NR | NR |
| Bhatla, 2020 | 50.00 | 55.00 | 13.00 | 50.00 | 26.00 | NR | 11.00 | 31.00 | NR | NR | NR | NR |
| Arabadjian, 2021 | 62.00 | 44.30 | NR | 64.30 | 18.60 | NR | 24.30 | 31.60 | 31.40 | 82.90 | 20.00 | NR |
| Amat-Santos, 2020 | 86.00 | 45.50 | NR | 54.50 | 18.20 | 9.10 | 18.20 | NR | NR | NR | NR | NR |
| Berrill, 2020 | 65.2 | 56 | 12.5 | 56.3 | 36.2 | NR | NR | NR | NR | NR | NR | NR |
| Mendes, 2020 | 86.3 | 56.6 | 28.1 | 71.5 | 23 | NR | 14.5 | NR | NR | NR | NR | NR |
| Quisi, 2020 | 55 | 44.7 | 2.3 | 32.5 | 29.9 | NR | 11.2 | NR | NR | NR | NR | NR |
| Rodilla, 2020 | 67.5 | 42.6 | 7.1 | 50.9 | 19.1 | NR | 8 | NR | NR | NR | NR | NR |
| Shi, 2020 | 63 | 52 | 3.3 | 29.7 | 14.5 | NR | 8.9 | NR | NR | NR | NR | NR |
| Rossi, 2020 | 76.2 | 32.4 | NR | 34.7 | 23.2 | NR | 16.1 | NR | NR | NR | NR | NR |
| Cho, 2020 | 70.3 | 38.5 | 18.2 | 55.2 | 35 | NR | 18.9 | NR | NR | NR | NR | NR |
| Gomez Antúnez1, 2020 | 69 | 43.3 | 7.5 | 50.4 | 18.7 | 7.2 | NR | NR | 15.4 | NR | 19.5 | NR |
| Denegri, 2020 | 65.6 | 34.6 | NR | 54.4 | 16.5 | NR | 13.3 | NR | NR | NR | NR | NR |
| Aajal, 2021 | 55.30 | 63.00 | NR | NR | NR | NR | NR | NR | NR | NR | NR | NR |
| Colon, 2020 | 64.60 | 32.00 | NR | NR | NR | NR | 16.00 | NR | NR | NR | 26.00 | NR |
| Iacopino, 2020 | 75.20 | 33.00 | 20.00 | 87.00 | 37.00 | NR | NR | 26.40 | NR | 3.00 | 29.00 | NR |
| Saleh, 2020 | 58.50 | 42.80 | 7.50 | 60.20 | 32.30 | NR | 11.40 | 28.20 | NR | 16.90 | 1.50 | NR |
| Russo, 2020 | 65.54 | 39.70 | 8.50 | 60.20 | 24.60 | NR | 13.20 | NR | NR | 13.40 | NR | NR |
| Peltzer, 2020 | 62.00 | 39.60 | NR | 51.20 | 29.70 | NR | 12.60 | NR | NR | NR | NR | NR |
| Oates, 2020 | 69.00 | 45.00 | 6.00 | 66.00 | 12.00 | NR | 19.00 | NR | NR | 30.00 | 15.00 | 17.00 |
| Mountantonakis, 2021 | 73.10 | 37.50 | 19.10 | 75.90 | 44.30 | NR | NR | NR | NR | NR | NR | NR |
| Linschoten, 2020 | 67.00 | 37.20 | NR | 44.60 | 23.10 | NR | NR | 28.10 | NR | NR | NR | NR |
| Kelesoglu, 2020 | 54.00 | 43.00 | 6.40 | 30.00 | 17.90 | NR | 14.50 | NR | NR | NR | NR | NR |
| Abe, 2020 | 58.00 | 45.30 | 12.70 | 60.60 | NR | NR | 14.10 | NR | NR | NR | NR | NR |
| Angeli, 2020 | 64.00 | 28.00 | 6.00 | 50.00 | 12.00 | NR | 10.00 | NR | NR | NR | NR | NR |
| Diandrea, 2020 | 66.60 | 40.00 | NR | NR | NR | NR | NR | 29.30 | NR | NR | NR | NR |
| Lanza, 2020 | 65.90 | 34.00 | NR | 52.20 | 11.40 | NR | 20.70 | NR | NR | NR | NR | NR |
| Li, 2020 | 64.00 | 48.90 | 8.10 | 32.60 | 14.80 | NR | 54.80 | NR | 4.40 | NR | 9.60 | NR |
| Maeda, 2020 | 64.00 | 45.70 | NR | 53.50 | 29.10 | NR | 10.00 | 28.70 | NR | NR | 15.00 | 15.10 |
| Musikantow, 2021 | 66.00 | 42.40 | NR | 34.40 | 24.60 | NR | NR | 27.80 | NR | NR | NR | NR |
| Piroth, 2020 | 65.00 | 47.00 | 8.00 | 33.10 | 19.00 | NR | NR | NR | NR | NR | NR | NR |
| Wetterslev, 2021 | 66.00 | 27.00 | NR | 44.00 | 21.00 | NR | NR | NR | 21.00 | 19.00 | 32.00 | NR |
| Atkins, 2020 | 74.30 | 23.50 | NR | 59.60 | 19.90 | NR | 21.50 | NR | NR | NR | NR | NR |
| Wang, 2020 | 64.97 | 52.40 | NR | 43.60 | 22.90 | NR | 14.70 | NR | NR | NR | NR | NR |
| Reilev, 2020 | 48.00 | 58.00 | 2.80 | 24.00 | 7.90 | NR | 7.70 | NR | 7.70 | 7.80 | NR | NR |
| Gerwen, 2020 | 56.80 | NR | 7.90 | NR | 28.20 | NR | NR | NR | NR | NR | NR | NR |
| Peterson, 2020 | 72.22 | 57.00 | 44.00 | 95.00 | 58.00 | NR | NR | NR | NR | NR | NR | NR |
| Ghio, 2020 | 68.60 | 29.30 | NR | NR | 20.00 | NR | 63.70 | NR | 34.00 | 34.60 | 45.10 | NR |
| Clift, 2020 | 48.21 | 6.49 | 1.17 | NR | NR | NR | NR | NR | NR | NR | NR | NR |
| Shah, 2020 | 78.40 | 36.73 | NR | 77.55 | NR | NR | NR | 28.70 | NR | NR | NR | NR |
| Alvarez-Garcia, 2020 | 63.50 | 44.90 | NR | 34.50 | 22.80 | NR | 14.00 | NR | 27.90 | 27.70 | 29.90 | NR |
| Canevelli, 2020 | 77.00 | 29.60 | 16.30 | 68.40 | 32.10 | NR | NR | NR | NR | NR | NR | NR |
| Izurieta, 2020 | NR | 55.60 | 8.00 | 58.30 | 23.50 | 0.30 | NR | NR | NR | NR | NR | NR |
| Polverino, 2020 | NR | 31.70 | NR | 47.20 | 16.30 | NR | 11.30 | NR | NR | NR | NR | NR |
| Russo, 2022 | 66.88 | 37.00 | 7.50 | 62.00 | 26.30 | NR | NR | NR | NR | NR | NR | NR |
| Rosenblatt,2022 | 73.00 | 40.00 | NR | 73.00 | 38.20 | NR | 33.00 | NR | NR | NR | NR | NR |
| Pillarisett，2022 | 68.00 | 45.00 | NR | NR | 38.00 | NR | 23.00 | NR | NR | 56.00 | NR | 25.00 |
| Lim,2023 | 65.00 | 42.00 | NR | 34.00 | 19.80 | NR | NR | NR | NR | NR | NR | NR |
| Li,2023 | 76.00 | 45.00 | NR | 56.00 | 25.00 | NR | 14.00 | NR | NR | NR | NR | NR |
| Fumagalli, 2022 | 82.00 | 48.00 | NR | NR | NR | NR | NR | NR | NR | NR | NR | NR |

BMI: Body Mass Index; DM: Diabetes; HF: heart failure; HBP: hypertension; TIA: transient ischemic attack; CVD: cardiovascular disease; ACEI: Angiotensin-Converting Enzyme Inhibitors; ARB: Angiotensin Receptor Blockers; NR: not reported.

# Table S5. Quality scores of the included studies

| **Study** | **Sample population** | **Sample size** | **Participation rate** | **Outcomeassessment** | **Analytical methods to control for bias** | **Total score** |
| --- | --- | --- | --- | --- | --- | --- |
| Benjamin A, 2016 | 2 | 2 | 2 | 1 | 2 | 9 |
| Cheng, 2019 | 2 | 2 | 2 | 1 | 1 | 8 |
| Zhou, 2021 | 2 | 2 | 2 | 1 | 2 | 9 |
| Vergara, 2021 | 2 | 2 | 2 | 1 | 1 | 8 |
| Velilla-Alonso, 2021 | 2 | 2 | 2 | 1 | 2 | 9 |
| Turgay Yildirim, 2021 | 2 | 1 | 1 | 1 | 1 | 6 |
| Terlecki, 2021 | 2 | 2 | 2 | 1 | 2 | 9 |
| Sanz, 2021 | 2 | 2 | 2 | 1 | 1 | 8 |
| Saleh, 2021 | 2 | 2 | 2 | 1 | 2 | 9 |
| Qureshi, 2021 | 2 | 2 | 2 | 1 | 1 | 8 |
| Puttegowda, 2021 | 2 | 2 | 2 | 1 | 2 | 9 |
| Phelps, 2021 | 2 | 2 | 2 | 1 | 1 | 8 |
| Paris, 2021 | 2 | 2 | 2 | 1 | 2 | 9 |
| Palmieri, 2020 | 2 | 2 | 2 | 1 | 1 | 8 |
| Pagnano, 2021 | 2 | 2 | 2 | 1 | 2 | 9 |
| Ozdemir, 2021 | 2 | 2 | 2 | 1 | 1 | 8 |
| Omar, 2021 | 2 | 2 | 2 | 1 | 2 | 9 |
| Mesquita, 2021 | 2 | 2 | 1 | 1 | 1 | 8 |
| Mathew, 2021 | 2 | 1 | 1 | 1 | 1 | 6 |
| Lee, 2021 | 2 | 1 | 1 | 1 | 1 | 6 |
| Lazcano, 2021 | 2 | 2 | 2 | 1 | 2 | 9 |
| Lala, 2020 | 2 | 2 | 2 | 1 | 1 | 8 |
| Kuno, 2022 | 2 | 2 | 2 | 1 | 2 | 9 |
| Kragholm, 2021 | 2 | 2 | 2 | 1 | 1 | 8 |
| Koutroumpakis, 2021 | 2 | 2 | 2 | 1 | 2 | 9 |
| Kanthasamy, 2021 | 2 | 2 | 2 | 1 | 1 | 8 |
| Inciardi, 2020 | 2 | 2 | 2 | 1 | 2 | 9 |
| Henein, 2021 | 2 | 2 | 2 | 1 | 1 | 8 |
| Harrison, 2021 | 2 | 2 | 2 | 1 | 2 | 9 |
| Garcia-Granja, 2021 | 2 | 2 | 2 | 1 | 1 | 8 |
| Fumagalli, 2021 | 2 | 2 | 2 | 1 | 2 | 9 |
| Ergün, 2020 | 2 | 2 | 2 | 1 | 1 | 8 |
| Chanseaume, 2021 | 2 | 2 | 2 | 1 | 2 | 9 |
| Bhatla, 2020 | 2 | 2 | 2 | 1 | 1 | 8 |
| Arabadjian, 2021 | 2 | 2 | 2 | 1 | 2 | 9 |
| Amat-Santos, 2020 | 2 | 2 | 2 | 1 | 1 | 8 |
| Berrill, 2020 | 2 | 1 | 1 | 1 | 1 | 6 |
| Mendes, 2020 | 2 | 2 | 2 | 1 | 1 | 8 |
| Quisi, 2020 | 2 | 2 | 2 | 1 | 2 | 9 |
| Rodilla, 2020 | 2 | 2 | 2 | 1 | 1 | 8 |
| Shi, 2020 | 2 | 2 | 2 | 1 | 2 | 9 |
| Rossi, 2020 | 2 | 2 | 2 | 1 | 1 | 8 |
| Cho, 2020 | 2 | 2 | 2 | 1 | 2 | 9 |
| Gomez Antunez1, 2020 | 2 | 2 | 2 | 1 | 1 | 8 |
| Denegri, 2020 | 2 | 2 | 2 | 1 | 2 | 9 |
| Aajal, 2021 | 2 | 2 | 2 | 1 | 1 | 8 |
| Colon, 2020 | 2 | 2 | 2 | 1 | 2 | 9 |
| Iacopino, 2020 | 2 | 1 | 1 | 1 | 1 | 6 |
| Saleh, 2020 | 2 | 2 | 2 | 1 | 2 | 9 |
| Russo, 2020 | 2 | 2 | 2 | 1 | 1 | 8 |
| Peltzer, 2020 | 2 | 2 | 2 | 1 | 2 | 9 |
| Oates, 2020 | 2 | 2 | 2 | 1 | 1 | 8 |
| Mountantonakis, 2021 | 2 | 2 | 2 | 1 | 2 | 9 |
| Linschoten, 2020 | 2 | 2 | 2 | 1 | 1 | 8 |
| Kelesoglu, 2020 | 2 | 2 | 2 | 1 | 2 | 9 |
| Abe, 2020 | 2 | 2 | 1 | 1 | 1 | 7 |
| Angeli, 2020 | 2 | 1 | 1 | 1 | 1 | 6 |
| Diandrea, 2020 | 2 | 2 | 2 | 1 | 1 | 8 |
| Lanza, 2020 | 2 | 2 | 2 | 1 | 2 | 9 |
| Li, 2020 | 2 | 2 | 2 | 1 | 1 | 8 |
| Maeda, 2020 | 2 | 2 | 2 | 1 | 2 | 9 |
| Musikantow, 2021 | 2 | 2 | 2 | 1 | 1 | 8 |
| Piroth, 2020 | 2 | 2 | 2 | 1 | 2 | 9 |
| Wetterslev, 2021 | 2 | 2 | 2 | 1 | 1 | 8 |
| Atkins, 2020 | 2 | 2 | 2 | 1 | 2 | 9 |
| Wang, 2020 | 2 | 2 | 2 | 1 | 1 | 8 |
| Reilev, 2020 | 2 | 2 | 2 | 1 | 2 | 9 |
| Gerwen, 2020 | 2 | 2 | 2 | 1 | 1 | 8 |
| Peterson, 2020 | 2 | 2 | 2 | 1 | 2 | 9 |
| Ghio, 2020 | 2 | 2 | 2 | 1 | 1 | 8 |
| Clift, 2020 | 2 | 2 | 2 | 1 | 2 | 9 |
| Shah, 2020 | 2 | 2 | 2 | 1 | 1 | 8 |
| Alvarez-Garcia, 2020 | 2 | 2 | 2 | 1 | 2 | 9 |
| Canevelli, 2020 | 2 | 2 | 2 | 1 | 1 | 8 |
| Izurieta, 2020 | 2 | 2 | 2 | 1 | 2 | 9 |
| Polverino, 2020 | 2 | 2 | 2 | 1 | 1 | 8 |
| Russo, 2022 | 2 | 2 | 2 | 1 | 2 | 9 |
| Rosenblatt,2022 | 2 | 2 | 2 | 1 | 1 | 8 |
| Pillarisett，2022 | 2 | 2 | 2 | 1 | 2 | 9 |
| Lim,2023 | 2 | 2 | 2 | 1 | 1 | 8 |
| Li,2023 | 2 | 2 | 2 | 1 | 2 | 9 |
| Fumagalli, 2022 | 2 | 2 | 2 | 1 | 1 | 8 |

# Table S6. Sensitivity analysis of pre-existing and new-onset AF rate

| Sensitivity analysis of pre-existing AF rate | | | | | |
| --- | --- | --- | --- | --- | --- |
| **Study omitted** | **Prevalence (95%CI)** | **Study omitted** | **Prevalence (95%CI)** | **Study omitted** | **Prevalence (95%CI)** |
| Zhou, 2021 | 0.10 (0.09, 0.12) | Lee, 2021 | 0.10 (0.09, 0.12) | Rossi, 2020 | 0.10 (0.09, 0.11) |
| Vergara, 2021 | 0.10 (0.09, 0.12) | Lazcano, 2021 | 0.10 (0.09, 0.12) | Cho, 2020 | 0.10 (0.09, 0.12) |
| Velilla-Alonso, 2021 | 0.10 (0.09, 0.11) | Lala, 2020 | 0.10 (0.09, 0.12) | Gomez Antunez1, 2020 | 0.10 (0.09, 0.12) |
| Turgay Yildirim, 2021 | 0.10 (0.09, 0.12) | Kuno, 2022 | 0.10 (0.09, 0.12) | Denegri, 2020 | 0.10 (0.09, 0.12) |
| Terlecki, 2021 | 0.10 (0.09, 0.12) | Kragholm, 2021 | 0.10 (0.09, 0.12) | Aajal, 2021 | 0.10 (0.09, 0.11) |
| Saleh, 2021 | 0.10 (0.09, 0.12) | Koutroumpakis, 2021 | 0.10 (0.09, 0.12) | Colon, 2020 | 0.10 (0.09, 0.12) |
| Qureshi, 2021 | 0.10 (0.09, 0.12) | Inciardi, 2020 | 0.10 (0.09, 0.12) | Iacopino, 2020 | 0.10 (0.09, 0.12) |
| Puttegowda, 2021 | 0.10 (0.09, 0.12) | Henein, 2021 | 0.10 (0.09, 0.12) | Saleh, 2020 | 0.10 (0.09, 0.12) |
| Phelps, 2021 | 0.10 (0.09, 0.12) | Harrison, 2021 | 0.10 (0.09, 0.12) | Russo, 2020 | 0.10 (0.09, 0.11) |
| Paris, 2021 | 0.10 (0.09, 0.12) | Fumagalli, 2021 | 0.10 (0.09, 0.12) | Peltzer, 2020 | 0.10 (0.09, 0.12) |
| Palmieri, 2020 | 0.10 (0.09, 0.11) | Chanseaume, 2021 | 0.10 (0.09, 0.12) | Oates, 2020 | 0.10 (0.09, 0.12) |
| Pagnano, 2021 | 0.10 (0.09, 0.11) | Bhatla, 2020 | 0.10 (0.09, 0.12) | Mountantonakis, 2021 | 0.10 (0.09, 0.11) |
| Ozdemir, 2021 | 0.10 (0.09, 0.12) | Arabadjian, 2021 | 0.10 (0.09, 0.11) | Linschoten, 2020 | 0.10 (0.09, 0.12) |
| Omar, 2021 | 0.10 (0.09, 0.12) | Amat-Santos, 2020 | 0.10 (0.09, 0.12) | Kelesoglu, 2020 | 0.10 (0.09, 0.12) |
| Mesquita, 2021 | 0.10 (0.09, 0.11) | Berrill, 2020 | 0.10 (0.09, 0.12) | Abe, 2020 | 0.10 (0.09, 0.12) |
| Mathew, 2021 | 0.10 (0.09, 0.12) | Mendes, 2020 | 0.10 (0.09, 0.11) | Angeli, 2020 | 0.10 (0.09, 0.12) |
| Maeda, 2020 | 0.10 (0.09, 0.12) | Quisi, 2020 | 0.10 (0.09, 0.12) | Diandrea, 2020 | 0.10 (0.09, 0.12) |
| Musikantow, 2021 | 0.10 (0.09, 0.12) | Rodilla, 2020 | 0.10 (0.09, 0.12) | Lanza, 2020 | 0.10 (0.09, 0.12) |
| Piroth, 2020 | 0.10 (0.09, 0.12) | Shi, 2020 | 0.10 (0.09, 0.12) | Li, 2020 | 0.10 (0.09, 0.12) |
| Wetterslev, 2021 | 0.10 (0.09, 0.11) | Gerwen, 2020 | 0.10 (0.09, 0.12) | Shah, 2020 | 0.10 (0.09, 0.12) |
| Atkins, 2020 | 0.10 (0.09, 0.12) | Peterson, 2020 | 0.10 (0.09, 0.12) | Alvarez-Garcia, 2020 | 0.10 (0.09, 0.12) |
| Wang, 2020 | 0.10 (0.09, 0.12) | Ghio, 2020 | 0.10 (0.09, 0.12) | Canevelli, 2020 | 0.10 (0.09, 0.11) |
| Reilev, 2020 | 0.10 (0.09, 0.12) | Clift, 2020 | 0.10 (0.09, 0.12) | Izurieta, 2020 | 0.09 (0.08, 0.09) |
| Polverino, 2020 | 0.10 (0.09, 0.12) | Russo, 2022 | 0.10 (0.09, 0.12) | Pillarisett，2022 | 0.10 (0.09, 0.12) |
| Li,2023 | 0.10 (0.09, 0.11) | Rosenblatt,2022 | 0.10 (0.09, 0.12) | Lim,2023 | 0.10 (0.09, 0.11) |
| Fumagalli, 2022 | 0.10 (0.09, 0.12) |  |  |  |  |
| **Sensitivity analysis of new-onset AF rate** | | | | | |
| **Study omitted** | **Prevalence (95%CI)** | **Study** | **Prevalence (95%CI)** | **Study** | **Prevalence (95%CI)** |
| Sanz, 2021 | 0.12 (0.09, 0.14) | Russo, 2020 | 0.10 (0.04, 0.15) | Kanthasamy, 2021 | 0.09 (0.04, 0.15) |
| Garcia-Granja, 2021 | 0.10 (0.04, 0.16) | Ergun, 2020 | 0.09 (0.04, 0.15) | Saleh, 2020 | 0.10 (0.04, 0.17) |
| Rosenblatt,2022 | 0.10 (0.04, 0.15) |  |  |  |  |

CI: confidence interval.

# Table S7. Sensitivity analysis of pre-existing and new-onset AF rate by regions

| **Sensitivity analysis of pre-existing and new-onset AF rate in Asia** | | | |
| --- | --- | --- | --- |
| **Study omitted** | **Prevalence (95%CI)** | **Study omitted** | **Prevalence (95%CI)** |
| Zhou, 2021 | 0.04 (0.03, 0.05) | Mathew, 2021 | 0.03 (0.02, 0.04) |
| Turgay Yildirim,2021 | 0.04 (0.03, 0.04) | Lee, 2021 | 0.04 (0.03, 0.05) |
| Saleh, 2021 | 0.03 (0.03, 0.04) | Quisi, 2020 | 0.03(0.02, 0.04) |
| Puttegowda, 2021 | 0.03 (0.02, 0.04) | Shi, 2020 | 0.04 (0.03, 0.05) |
| Ozdemir, 2021 | 0.03 (0.03, 0.04) | Kelesoglu, 2020 | 0.03 (0.02, 0.04) |
| Omar, 2021 | 0.03 (0.02, 0.04) | Li, 2020 | 0.03 (0.02, 0.04) |
| Wang, 2020 | 0.03 (0.02, 0.04) | Lim,2023 | 0.03 (0.02, 0.04) |
| **Sensitivity analysis of pre-existing and new-onset AF rate in North America** | | | |
| **Study omitted** | **Prevalence (95%CI)** | **Study omitted** | **Prevalence (95%CI)** |
| Qureshi, 2021 | 0.10 (0.08, 0.12) | Peltzer, 2020 | 0.10 (0.09, 0.11) |
| Pagnano, 2021 | 0.10 (0.09, 0.11) | Oates, 2020 | 0.10 (0.09, 0.11) |
| Kuno, 2022 | 0.10 (0.09, 0.12) | Mountantonakis, 2021 | 0.10 (0.09, 0.11) |
| Koutroumpakis, 2021 | 0.10 (0.09, 0.11) | Abe, 2020 | 0.10 (0.09, 0.11) |
| Bhatla, 2020 | 0.10 (0.09, 0.12) | Maeda, 2020 | 0.10 (0.09, 0.11) |
| Arabadjian, 2021 | 0.10 (0.09, 0.11) | Musikantow, 2021 | 0.10 (0.09, 0.11) |
| Cho, 2020 | 0.10 (0.09, 0.11) | Gerwen, 2020 | 0.10 (0.09, 0.11) |
| Colon, 2020 | 0.10 (0.09, 0.11) | Peterson, 2020 | 0.10 (0.09, 0.11) |
| Saleh, 2020 | 0.10 (0.09, 0.11) | Alvarez-Garcia, 2020 | 0.10 (0.09, 0.11) |
| Izurieta, 2020 | 0.10 (0.08, 0.12) | Rosenblatt,2022 | 0.10 (0.09, 0.12) |
| Pillarisett,2022 | 0.10 (0.09, 0.11) |  |  |
| **Sensitivity analysis of pre-existing rate of AF in Europe** | | | |
| Vergara, 2021 | 0.10 (0.10, 0.11) | Rodilla, 2020 | 0.10 (0.10, 0.10) |
| Velilla-Alonso, 2021 | 0.10 (0.10, 0.11) | Rossi, 2020 | 0.10 (0.10, 0.11) |
| Terlecki, 2021 | 0.10 (0.09, 0.10) | Gomez Antunez1, 2020 | 0.10 (0.10, 0.10) |
| Phelps, 2021 | 0.10 (0.10, 0.11) | Denegri, 2020 | 0.10 (0.10, 0.11) |
| Paris, 2021 | 0.10 (0.10, 0.11) | Iacopino, 2020 | 0.10 (0.10, 0.11) |
| Palmieri, 2020 | 0.10 (0.10, 0.10) | Russo, 2020 | 0.10 (0.10, 0.10) |
| Mesquita, 2021 | 0.10 (0.10, 0.11) | Linschoten, 2020 | 0.10 (0.10, 0.11) |
| Lazcano, 2021 | 0.10 (0.10, 0.11) | Angeli, 2020 | 0.10 (0.10, 0.11) |
| Lala, 2020 | 0.10 (0.10, 0.11) | Diandrea, 2020 | 0.10 (0.10, 0.11) |
| Kragholm, 2021 | 0.10 (0.10, 0.11) | Lanza, 2020 | 0.10 (0.10, 0.11) |
| Inciardi, 2020 | 0.10 (0.10, 0.11) | Piroth, 2020 | 0.10 (0.10, 0.10) |
| Henein, 2021 | 0.10 (0.10, 0.11) | Wetterslev, 2021 | 0.10 (0.10, 0.11) |
| Harrison, 2021 | 0.10 (0.09, 0.11) | Atkins, 2020 | 0.10 (0.10, 0.11) |
| Fumagalli, 2021 | 0.10 (0.09, 0.11) | Reilev, 2020 | 0.10 (0.10, 0.11) |
| Chanseaume, 2021 | 0.13 (0.11, 0.14) | Ghio, 2020 | 0.10 (0.10, 0.11) |
| Amat-Santos, 2020 | 0.10 (0.10, 0.11) | Clift, 2020 | 0.13 (0.11, 0.14) |
| Berrill, 2020 | 0.10 (0.10, 0.11) | Canevelli, 2020 | 0.10 (0.09, 0.10) |
| Mendes, 2020 | 0.10 (0.10, 0.11) | Polverino, 2020 | 0.10 (0.10, 0.11) |
| Russo, 2022 | 0.10 (0.10, 0.11) | Li,2023 | 0.10 (0.10, 0.11) |
| Fumalli, 2022 | 0.10 (0.10, 0.11) |  |  |

# Table S8. Sensitivity analysis of effect of pre-existing AF on mortality.

| **Study omitted** | **Prevalence (95%CI)** | **Study omitted** | **Prevalence (95%CI)** |
| --- | --- | --- | --- |
| Izurieta, 2020 | 2.09 (1.56, 2.61) | Ghio, 2020 | 1.88 (1.50, 2.26) |
| Clift, 2020 | 2.06 (1.55, 2.57) | Mendes, 2020 | 1.94 (1.55, 2.34) |
| Rodilla, 2020 | 1.73 (1.39, 2.06) | Denegri, 2020 | 1.90 (1.52, 2.28) |
| Pagnano, 2021 | 1.63 (1.30, 1.96) | Cho, 2020 | 1.93 (1.54, 2.31) |
| Lee, 2021 | 1.84 (1.47, 2.22) | Inciardi, 2020 | 1.91 (1.53, 2.29) |
| Alvarez-Garcia, 2020 | 2.00 (1.58, 2.41) | Berrill, 2020 | 1.91 (1.53, 2.29) |
| Musikantow, 2021 | 1.89 (1.51, 2.27) | Shah, 2020 | 1.91 (1.52, 2.30) |
| Canevelli, 2020 | 1.99 (1.58, 2.40) | Russo, 2020 | 1.95 (1.55, 2.34) |
| Terlecki, 2021 | 1.85 (1.47, 2.24) | Peterson, 2020 | 2.01 (1.61, 2.41) |
| Peltzer, 2020 | 1.82 (1.44, 2.20) | Ozdemir, 2021 | 1.88 (1.50, 2.26) |
| Paris, 2021 | 1.89 (1.50, 2.28) | Quisi, 2020 | 1.89 (1.51, 2.27) |
| Shi, 2020 | 1.90 (1.52, 2.28) | Rossi, 2020 | 1.93 (1.54, 2.33) |
| Li,2023 | 1.98 (1.60, 2.28) |  |  |

# Table S9. Meta-regression of AF prevalence in COVID-19

| **Variables** | **No. of reported studies** | **β coefficient (95%CI)** | ***P* value** |
| --- | --- | --- | --- |
| Mean age | 78 | -0.0008 (1.001**–**1.002) | 0.001 |
| Female | 78 | -0.0017 (0.999**–**1.001) | 0.164 |
| HF | 47 | 0.0030 (1.000**–**1.001) | 0.924 |
| HBP | 67 | 0.0003 (0.999**–**1.001) | 0.243 |
| DM | 66 | -0.0021 (0.999**–**1.001) | 0.294 |
| TIA | 4 | 1.000 (0.990**–**1.020) | 0.658 |
| CVD | 38 | 1.000 (0.999**–**1.001) | 0.144 |
| BMI | 10 | 1.000 (0.995**–**1.001) | 0.509 |
| Co-antiplatelet agents | 14 | 1.001 (0.996**–**1.007) | 0.481 |
| Beta blocker agents | 18 | 1.001 (0.998**–**1.004) | 0.638 |
| co-ACEI | 17 | 1.001 (0.994**–**1.007) | 0.864 |
| co-ARB | 6 | 0.0598 (-0.217-0.337) | 0.581 |

BMI: Body Mass Index; DM: Diabetes; HF: heart failure; HBP: hypertension; TIA: transient ischemic attack; CVD: cardiovascular disease; ACEI: Angiotensin-Converting Enzyme Inhibitors; ARB: Angiotensin Receptor Blockers.

# Table S10. Meta-regression of effect of pre-existing and new-onset AF on mortality.

| **Variables** | **No. of reported studies** | **β coefficient (95%CI)** | ***P* value** |
| --- | --- | --- | --- |
| Mean age | 24 | 0.9674 (0.6289-1.4879) | 0.874 |
| Female | 25 | 0.1277 (-0.0397, 0.2950) | 0.128 |
| HF | 16 | 0.0133 (-0.0797, 0.1062) | 0.764 |
| HBP | 22 | 0.1763 (-0.1726, 0.5252) | 0.305 |
| DM | 21 | -0.1112 (-0.5302, 0.3077) | 0.585 |
| TIA | 3 | -0.2442 (-2.9329, 2.4446) | 0.455 |
| CVD | 13 | 0.0325 (-0.1207, 0.1857) | 0.650 |
| BMI | 3 | 0.1331 (-2.4788, 2.7451) | 0.634 |
| Co-antiplatelet agents | 4 | 0.0845 (-2.3152, 2.4842) | 0.893 |
| Beta blocker agents | 6 | 0.3578 (-0.5382, 1.2538) | 0.330 |
| co-ACEI | 4 | 0.0643 (-1.5870, 1.7157) | 0.882 |
| co-ARB | 3 | -0.0618 (-5.7190, 5.5954) | 0.912 |

BMI: Body Mass Index; DM: Diabetes; HF: heart failure; HBP: hypertension; TIA: transient ischemic attack; CVD: cardiovascular disease; ACEI: Angiotensin-Converting Enzyme Inhibitors; ARB: Angiotensin Receptor Blockers.

# Table S11. Detailed characteristics of the included studies

| **Study** | **Country /region** | **Study type** | **Data source** | **Outcomes type** | **Total number** | **AF**  **number** |
| --- | --- | --- | --- | --- | --- | --- |
| Zhou, 2021 | China | Retrospective | Hong Kong’s public hospitals | pre-existing AF | 4442 | 43 |
| Vergara, 2021 | Italy | Retrospective | Piacenza Hospital network | pre-existing AF | 1049 | 115 |
| Velilla-Alonso, 2021 | Spain | Retrospective | Hospital General Universitario Gregorio | pre-existing AF | 195 | 36 |
| Turgay Yildirim, 2021 | Turkey | Retrospective | Eskisehir City Hospital | pre-existing AF | 139 | 2 |
| Terlecki, 2021 | Poland | Retrospective | University Hospital in Kraków | pre-existing AF, mortality | 1729 | 233 |
| Sanz, 2021 | Spain | Retrospective | University Hospital Ramón y Cajal, | new-onset, mortality | 160 | 12 |
| Saleh, 2021 | Saudi Arabia | Retrospective | Saudi Arabia hospital | pre-existing AF | 723 | 20 |
| Qureshi, 2021 | USA | Retrospective | 62 health care facilities | pre-existing AF | 85491 | 8591 |
| Puttegowda, 2021 | India | Retrospective | Institute of Cardiovascular Sciences and Research | pre-existing AF | 511 | 24 |
| Phelps, 2021 | Denmark | Retrospective | Several national data registries in Denmark | pre-existing AF | 4090 | 479 |
| Paris, 2021 | Italy | Retrospective | Cardiology Units in Italy | pre-existing AF, mortality | 696 | 106 |
| Palmieri, 2020 | Italy | Retrospective | Provinces of Trento and Bozen | pre-existing AF | 3032 | 681 |
| Pagnano, 2021 | USA | Retrospective | 13 Northwell Health hospitals | pre-existing AF, mortality | 9564 | 1687 |
| Ozdemir, 2021 | Turkey | Retrospective | Manisa Merkezefendi State Hospital | pre-existing AF, mortality | 350 | 40 |
| Omar, 2021 | Turkey | Retrospective | Kars Harakani State Hospital | pre-existing AF | 453 | 13 |
| Mesquita, 2021 | Portugal | Retrospective | Portuguese hospitals | pre-existing AF | 64 | 19 |
| Mathew, 2021 | India | Retrospective | 13 hospitals from south India | pre-existing AF | 62 | 3 |
| Lee, 2021 | Korea | Retrospective | Dataset from the OpenData4Covid19 | pre-existing AF, mortality | 7162 | 130 |
| Lazcano, 2021 | Spain | Retrospective | Health administration databases | pre-existing AF | 91629 | 3600 |
| Lala, 2020 | Italy | Retrospective | Five Mount Sinai Health System hospitals | pre-existing AF | 2736 | 206 |
| Kuno, 2022 | USA | Retrospective | Mount Sinai Health System | pre-existing AF | 6095 | 381 |
| Kragholm, 2021 | Denmark | Retrospective | Nationwide register-based study | pre-existing AF | 4842 | 290 |
| Koutroumpakis, 2021 | USA | Retrospective | Electronic medical records | pre-existing AF | 514 | 37 |
| Kanthasamy, 2021 | England | Retrospective | St Bartholomew’s Hospital | new-onset, mortality | 109 | 16 |
| Inciardi, 2020 | Italy | Retrospective | Civil Hospitals of Brescia, | pre-existing AF, mortality | 99 | 19 |
| Henein, 2021 | UK | Retrospective | Electronic medical records | pre-existing AF | 748 | 81 |
| Harrison, 2021 | UK | Retrospective | Global federated health research network. | pre-existing AF | 68975 | 7109 |
| Garcia-Granja, 2021 | Spain | Retrospective | Ter-tiary hospital2020.3.10-2020.4.15 | new-onset, mortality | 517 | 54 |
| Fumagalli, 2021 | Italy | Retrospective | GeroCovid e-RegistryO | pre-existing AF | 806 | 176 |
| Ergun, 2020 | Turkey | Retrospective | Faculty of Medicine | new-onset, mortality | 248 | 37 |
| Chanseaume, 2021 | UK | Prospective | QResearch database linked to data | pre-existing AF | 6952440 | 222783 |
| Bhatla, 2020 | USA | Retrospective | University of Pennsylvania | pre-existing AF | 700 | 39 |
| Arabadjian, 2021 | USA | Retrospective | Large urban hospital system in the NewYork | pre-existing AF | 70 | 23 |
| Amat-Santos, 2020 | Spain | Retrospective | National, multicenter, open-label | pre-existing AF | 102 |  |
| Berrill, 2020 | UK | Retrospective | District general hospital | pre-existing AF, mortality | 48 | 7 |
| Mendes, 2020 | Switzerland | Retrospective | University Hospitals | pre-existing AF, mortality | 235 | 58 |
| Quisi, 2020 | Turkey | Retrospective | Okmeydanı Training and Research Hospital | pre-existing AF, mortality | 349 | 16 |
| Rodilla, 2020 | Spain | Retrospective | 150 hospitals from the 17 regions in Spain | pre-existing AF, mortality | 12226 | 6278 |
| Shi, 2020 | China | Retrospective | Renmin Hospital of Wuhan University | pre-existing AF, mortality | 671 | 7 |
| Rossi, 2020 | Italy | Retrospective | Piacenza Hospital network | pre-existing AF, mortality | 590 | 101 |
| Cho, 2020 | USA | Prospective | Cedars-Sinai Medical Center | pre-existing AF, mortality | 143 | 18 |
| Gomez Antunez1, 2020 | Spain | Retrospective | Hospitalized in 150 hospitals in Spain | pre-existing AF | 10395 | 1175 |
| Denegri, 2020 | Italy | Retrospective | Modena University Hospital | pre-existing AF, mortality | 201 | 12 |
| Aajal, 2021 | Morocco | Prospective | Centre hospitalier universitaire | pre-existing AF | 100 | 22 |
| Colon, 2020 | USA | Prospective | University of Alabama at Birmingham Hospital | pre-existing AF | 115 | 6 |
| Iacopino, 2020 | Italy | Prospective | Intensive Care Unit or the general Medicine | pre-existing AF | 30 | 8 |
| Saleh, 2020 | USA | Prospective | 3 hospitals within the Northwell Health system | pre-existing AF, new-onset AF | 201 | 14 |
| Russo, 2020 | Italy | Retrospective | Ten Italian Hospitals | pre-existing AF, new-onset AF | 414 | 72 |
| Peltzer, 2020 | USA | Retrospective | New York Presbyterian/Weill Cornell Medicine | pre-existing AF, mortality | 1053 | 154 |
| Oates, 2020 | USA | Retrospective | Mount Sinai Hospital | pre-existing AF | 77 | 4 |
| Mountantonakis, 2021 | USA | Retrospective | 13 Northwell Health hospitals | pre-existing AF | 9564 | 1687 |
| Linschoten, 2020 | Netherlands | Retrospective | International patient registry | pre-existing AF | 3011 | 142 |
| Kelesoglu, 2020 | Turkey | Retrospective | Kayseri City Hospital, | pre-existing AF | 658 | 33 |
| Abe, 2020 | USA | Retrospective | Grady Memorial Hospital | pre-existing AF | 142 | 9 |
| Angeli, 2020 | Italy | Retrospective | Maugeri Care and Research Institute of Tradate | pre-existing AF | 50 | 3 |
| Diandrea, 2020 | Italy | Retrospective | Umberto I, M.Scarlato, Cardarelli and Monaldi | pre-existing AF | 280 | 51 |
| Lanza, 2020 | Italy | Prospective | Emergency Department of University Hospital | pre-existing AF | 324 | 20 |
| Li, 2020 | China | Retrospective | Wuhan Asia General hospital | pre-existing AF | 135 | 8 |
| Maeda, 2020 | USA | Retrospective | Mount Sinai Beth Israel | pre-existing AF | 181 | 9 |
| Musikantow, 2021 | USA | Retrospective | 5 hospitals within the Mount Sinai Health System | pre-existing AF, mortality | 3970 | 375 |
| Piroth, 2020 | France | Retrospective | French national administrative database (PMSI) | pre-existing AF | 89530 | 11129 |
| Wetterslev, 2021 | Denmark | Retrospective | Capital Region of Denmark | pre-existing AF | 155 | 52 |
| Atkins, 2020 | UK | Retrospective | 22 assessment centers in England, Scotland | pre-existing AF | 507 | 68 |
| Wang, 2020 | China | Retrospective | Sino-French New City Campus of Tongji Hospital | pre-existing AF | 319 | 20 |
| Reilev, 2020 | Denmark | Retrospective | Nationwide data | pre-existing AF | 11122 | 603 |
| Gerwen, 2020 | USA | Retrospective | a large New York City health system | pre-existing AF | 3707 | 313 |
| Peterson, 2020 | USA | Retrospective | Einstein Medical Center Philadelphia hospital | pre-existing AF, mortality | 355 | 39 |
| Ghio, 2020 | Italy | Prospective | A cademic hospital in Lombardy | pre-existing AF, mortality | 340 | 25 |
| Clift, 2020 | UK | Prospective | New and Emerging Respiratory Virus Threats | pre-existing AF, mortality | 6083102 | 147528 |
| Shah, 2020 | USA | Retrospective | Community Medical Center | pre-existing AF, mortality | 487 | 74 |
| Alvarez-Garcia, 2020 | USA | Retrospective | 5 Mount Sinai Health System hospitals | pre-existing AF, mortality | 6439 | 464 |
| Canevelli, 2020 | Italy | Retrospective | Italian National Institute of Health | pre-existing AF, mortality | 2621 | 584 |
| Izurieta, 2020 | USA | Retrospective | Medicare fee-for-service (FFS) beneficiaries | pre-existing AF, mortality | 25333329 | 2888000 |
| Polverino, 2020 | Italy | Retrospective | Medical charts from 61 hospitals across Italy | pre-existing AF | 3179 | 256 |
| Russo, 2022 | Italy | Retrospective | 9 Italian Hospitals | pre-existing AF | 467 | 122 |
| Rosenblatt, 2022 | USA | Retrospective | COVID-19 Cardiovascular Disease Registry | pre-existing AF, new-onset AF | 36083 | 978 |
| Pillarisett, 2022 | USA | Retrospective | Electronic medical record data | pre-existing AF | 81844 | 3683 |
| Lim, 2023 | Korea | Retrospective | Health Insurance Review and Assessment Service | pre-existing AF, mortality | 107247 | 1919 |
| Terlecki, 2023 | Poland | Retrospective | University Hospital in Krakow | pre-existing AF, mortality | 4998 | 535 |
| Fumagalli, 2022 | Italy | Retrospective | GeroCovid Observational, multicenter registry | pre-existing AF | 808 | 176 |

AF: atrial fibrillation; USA: United States of America; UK: United Kingdom.


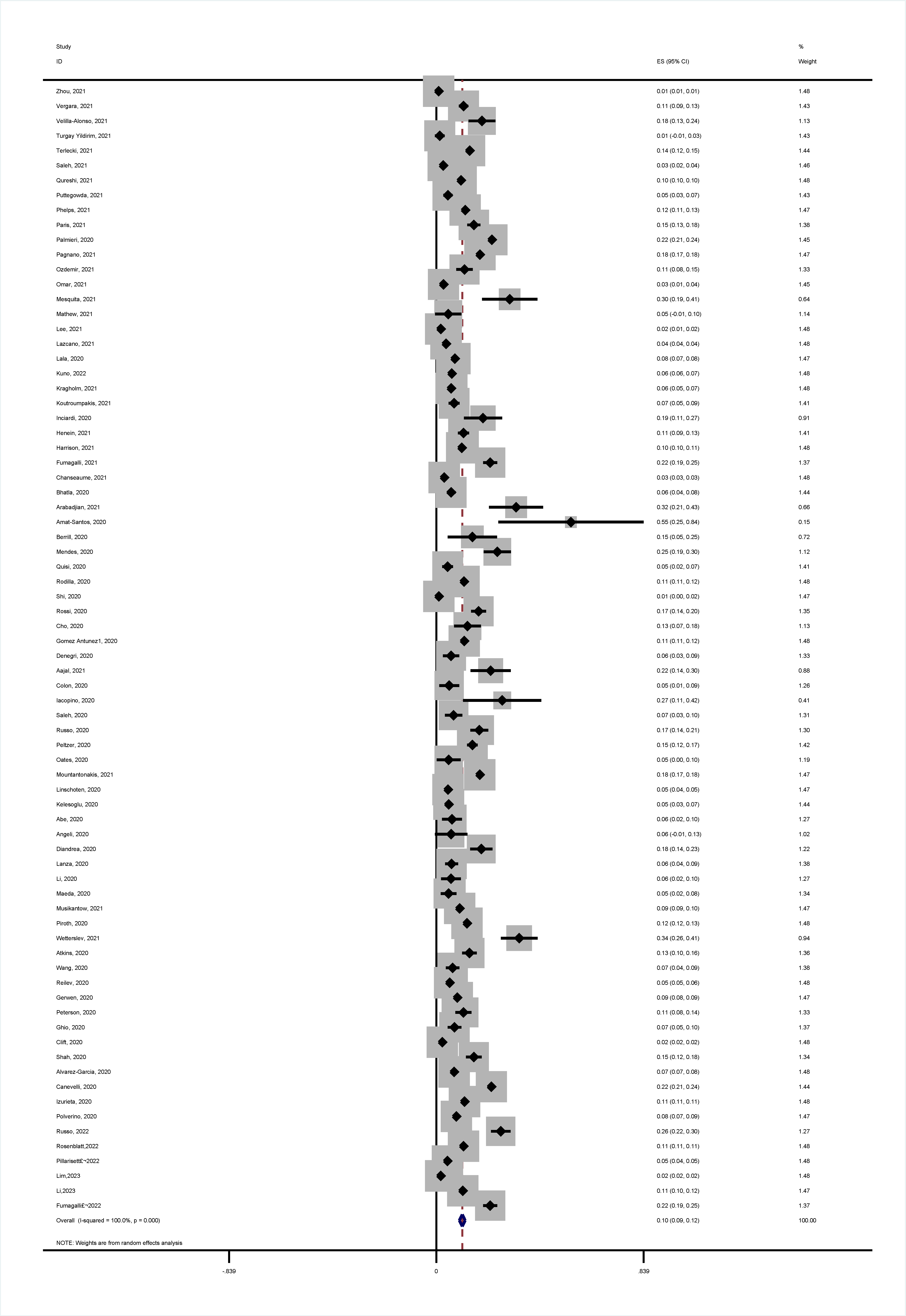


**Figure S1. Pooled prevalence of pre-existing rate of AF in COVID-19**

**
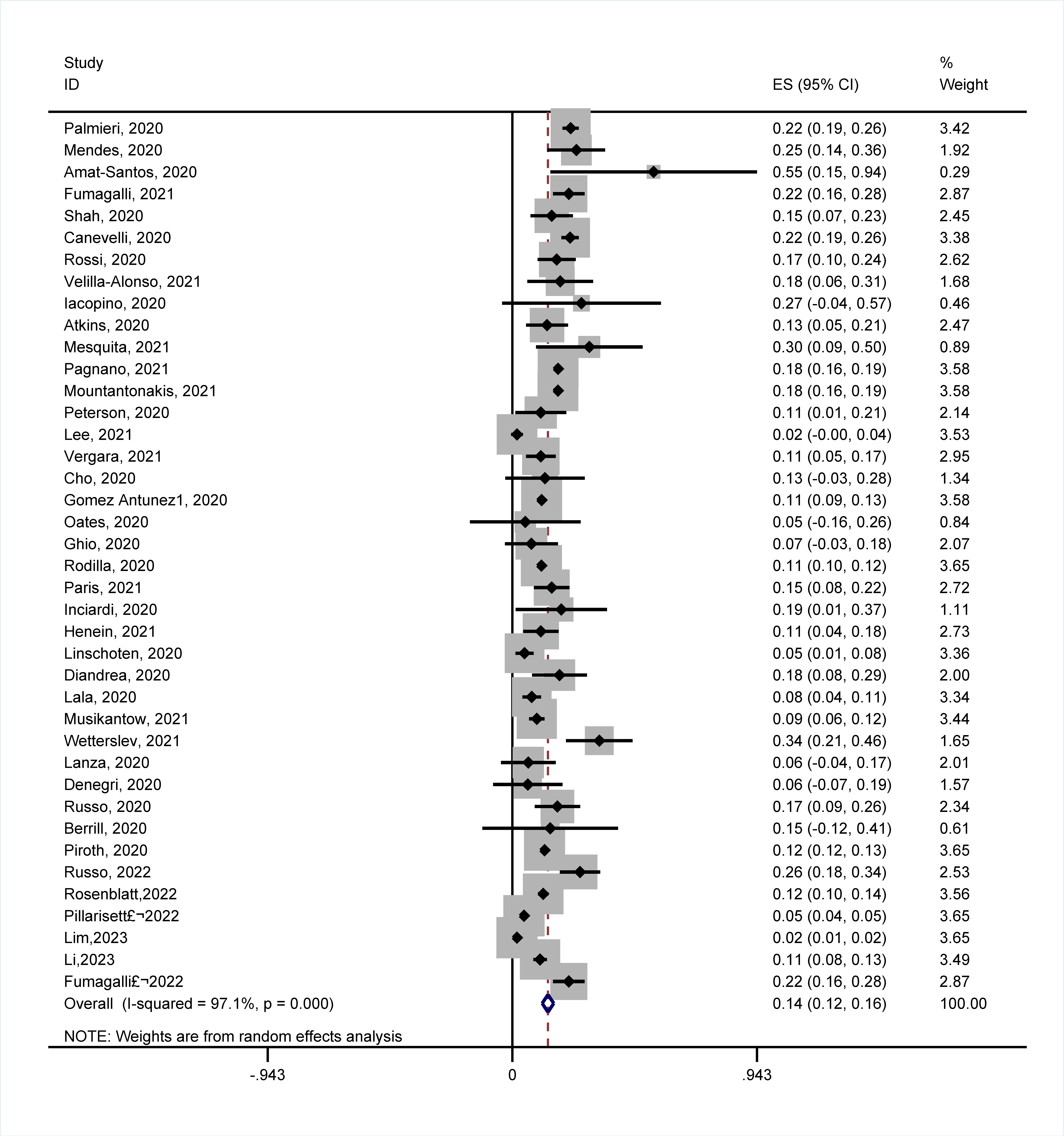
**

**Figure S2. Pooled prevalence of pre-existing rate of AF in mean age≥ 65 years**

**
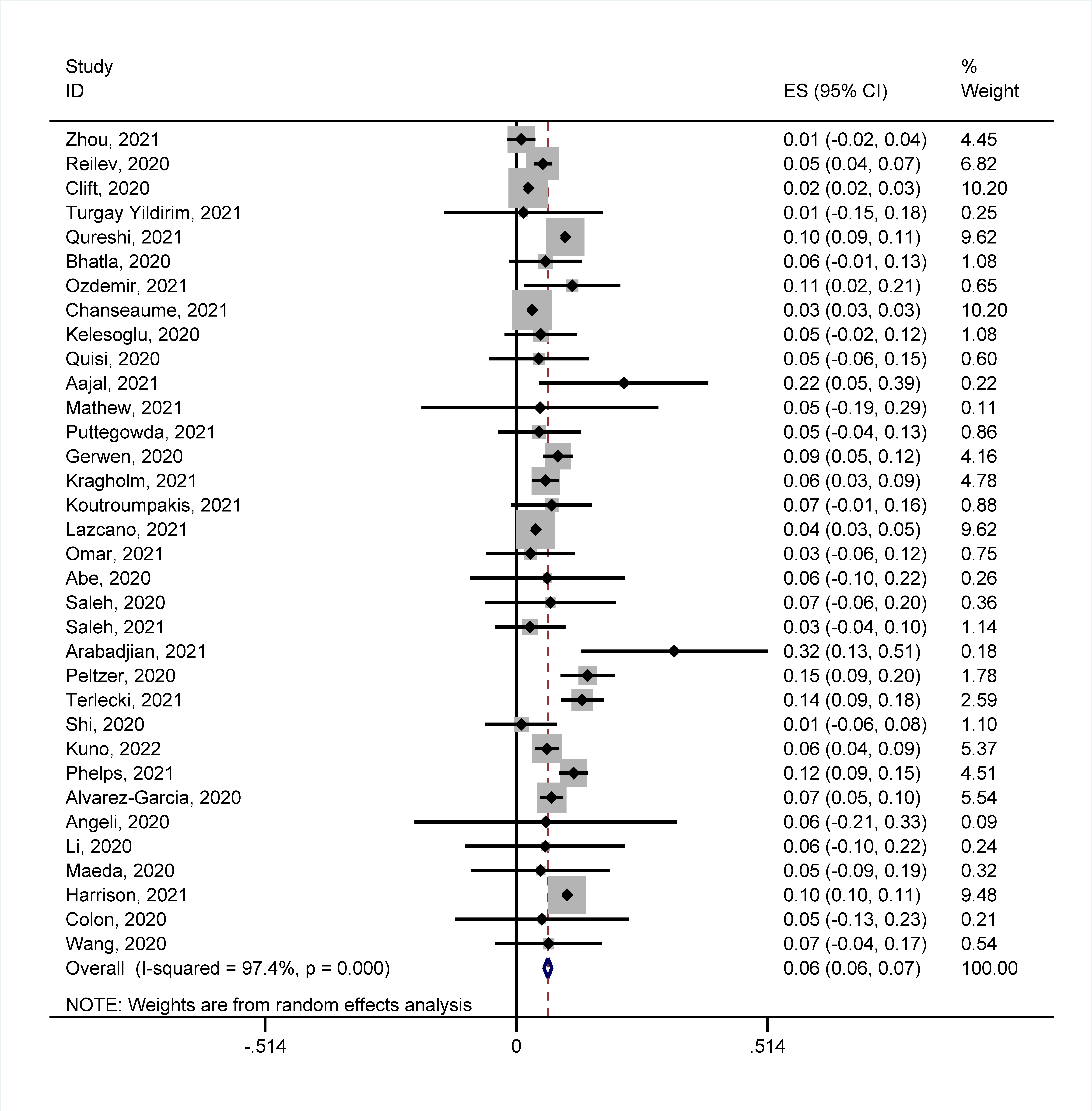
**

**Figure S3. Pooled prevalence of pre-existing rate of AF in mean age < 65 years**

**
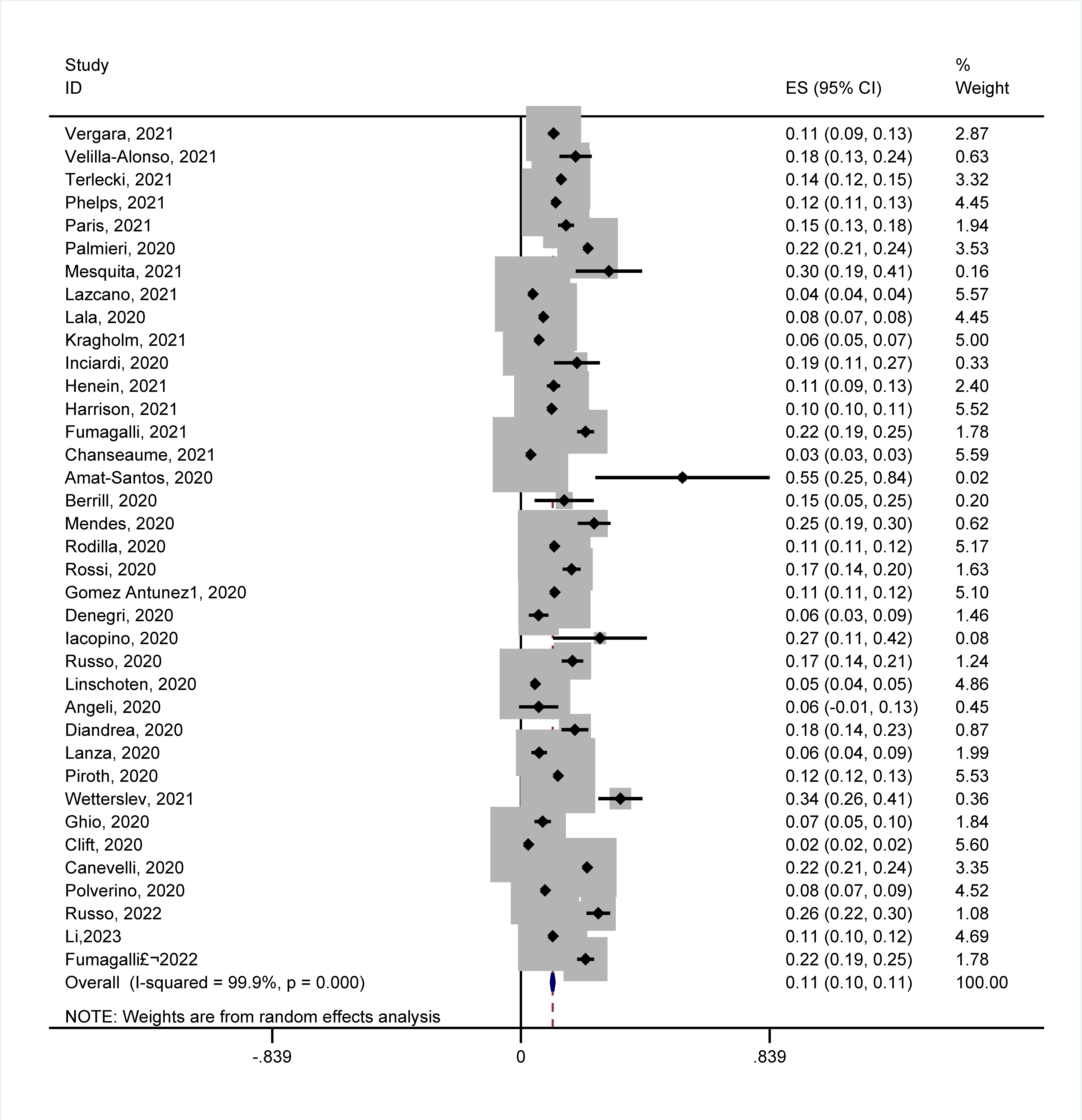
**

**Figure S4. Pooled prevalence of pre-existing rate of AF in COVID-19 in Europe**

**
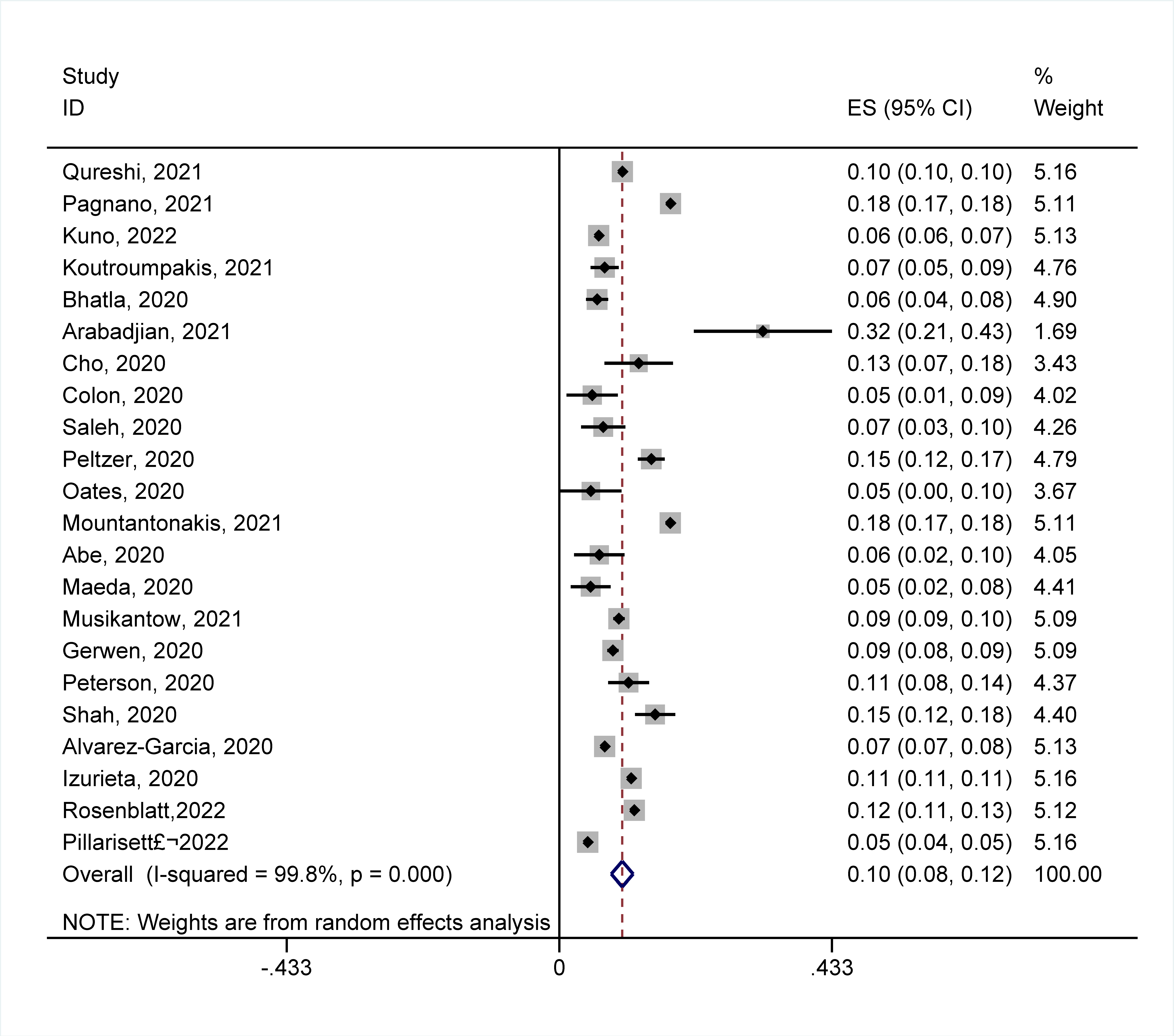
**

**Figure S5. Pooled prevalence of pre-existing rate of AF in COVID-19 in North America**

**
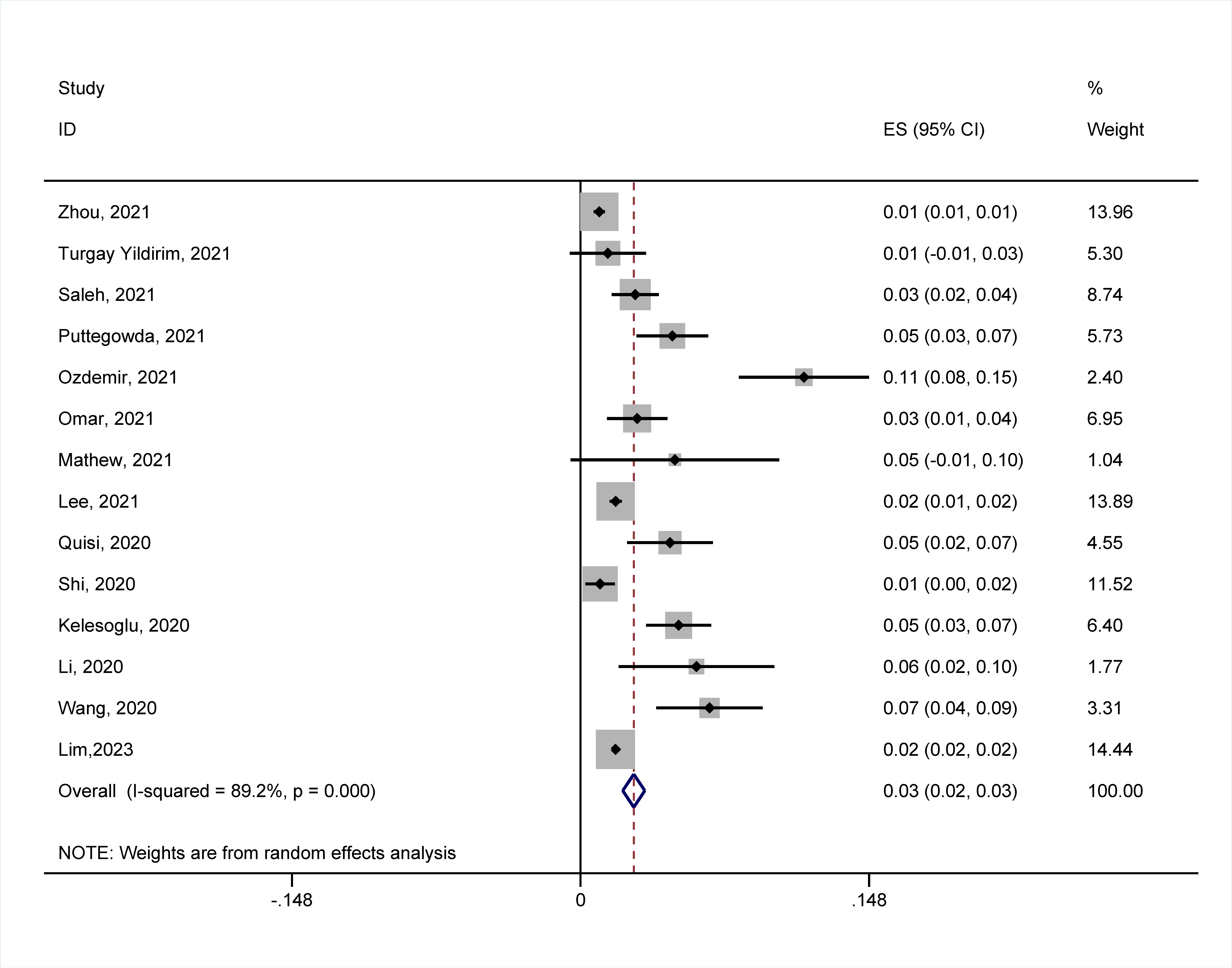
**

**Figure S6. Pooled prevalence of pre-existing rate of AF in COVID-19 in Asia**

**
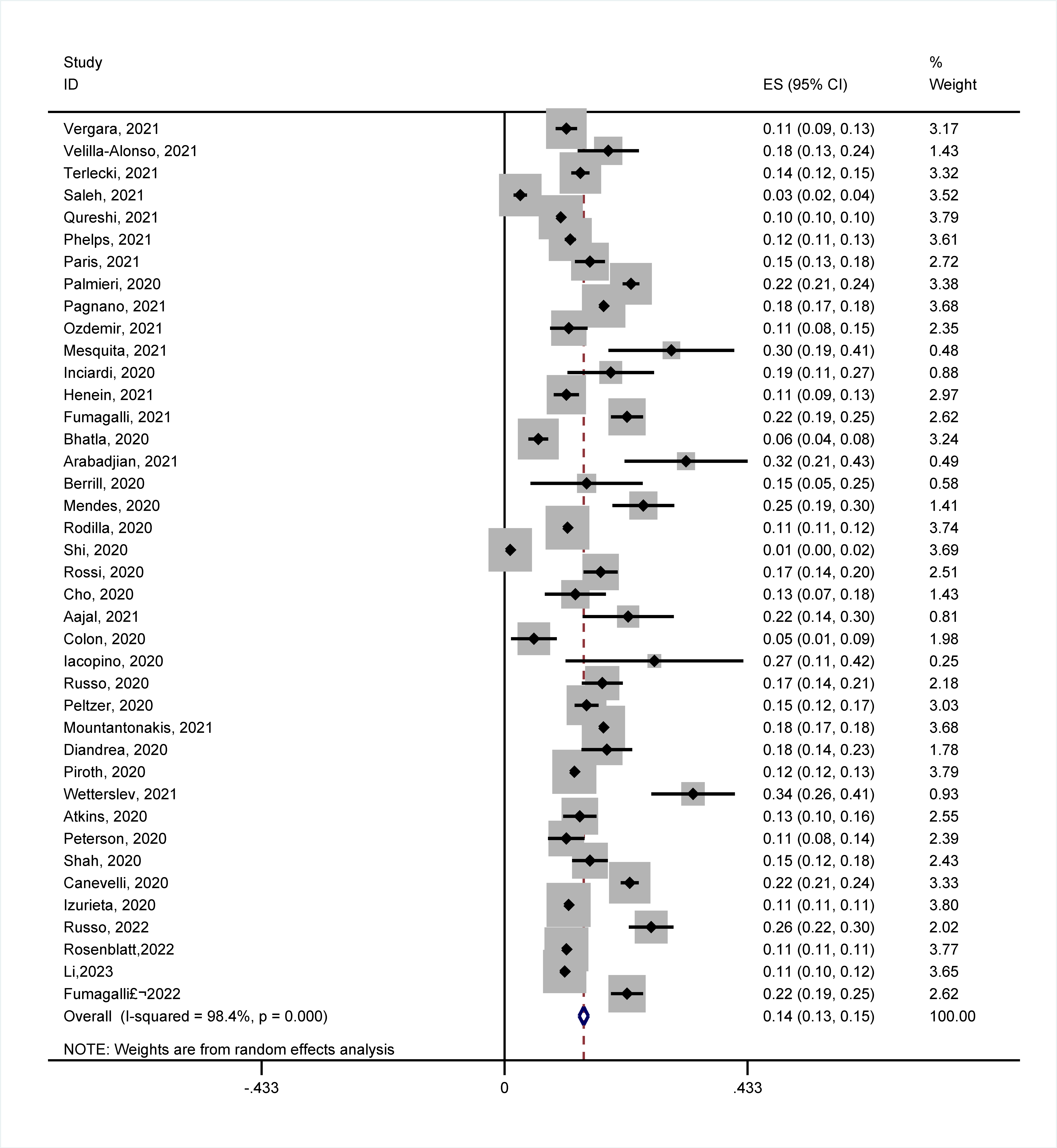
**

**Figure S7. Pooled prevalence of pre-existing rate of AF in severe patients**

**
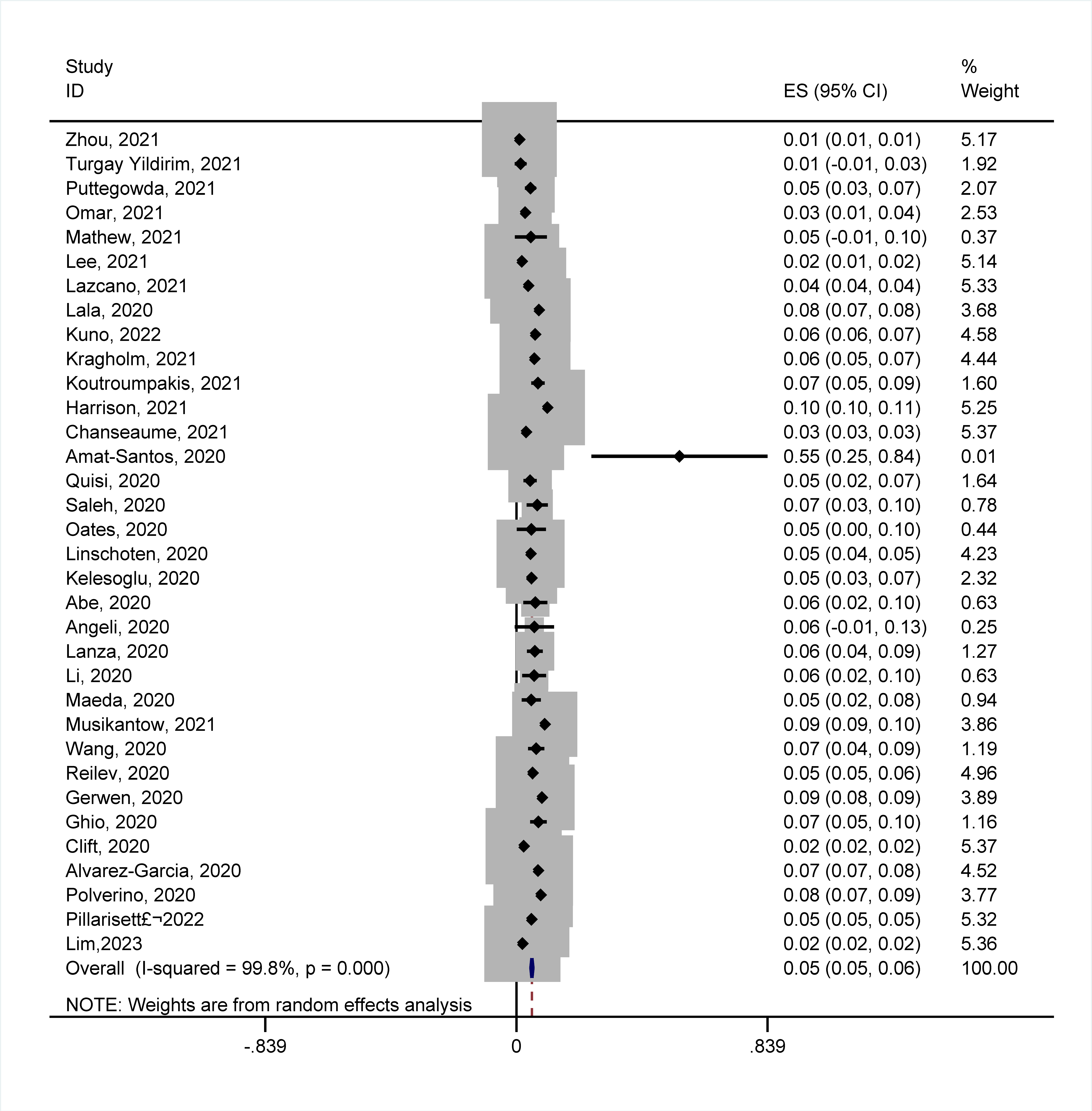
**

**Figure S8. Pooled prevalence of pre-existing rate of AF in non-severe patients**

**
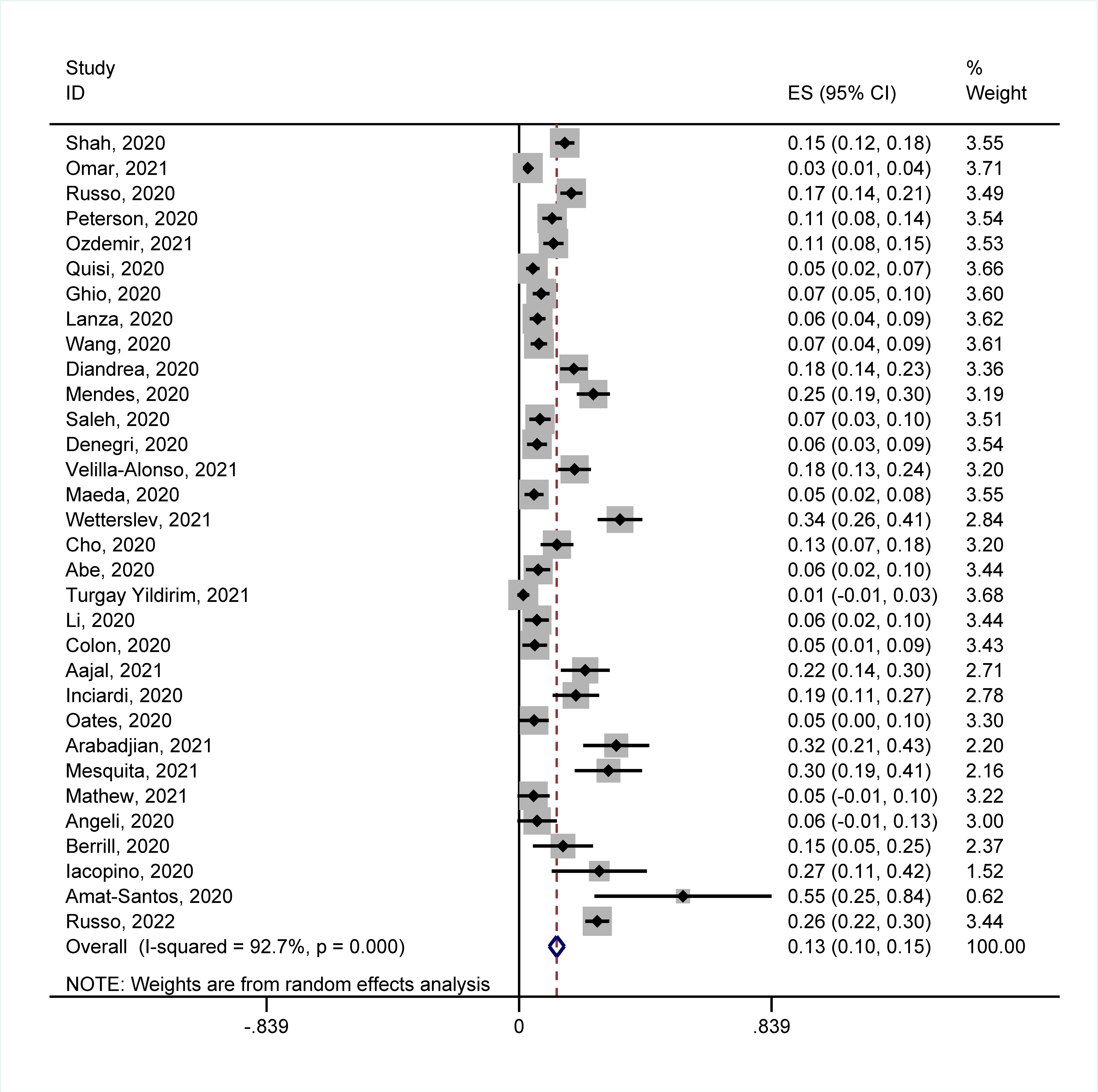
**

**Figure S9. Pooled prevalence of pre-existing rate of AF in sample size < 500**

**
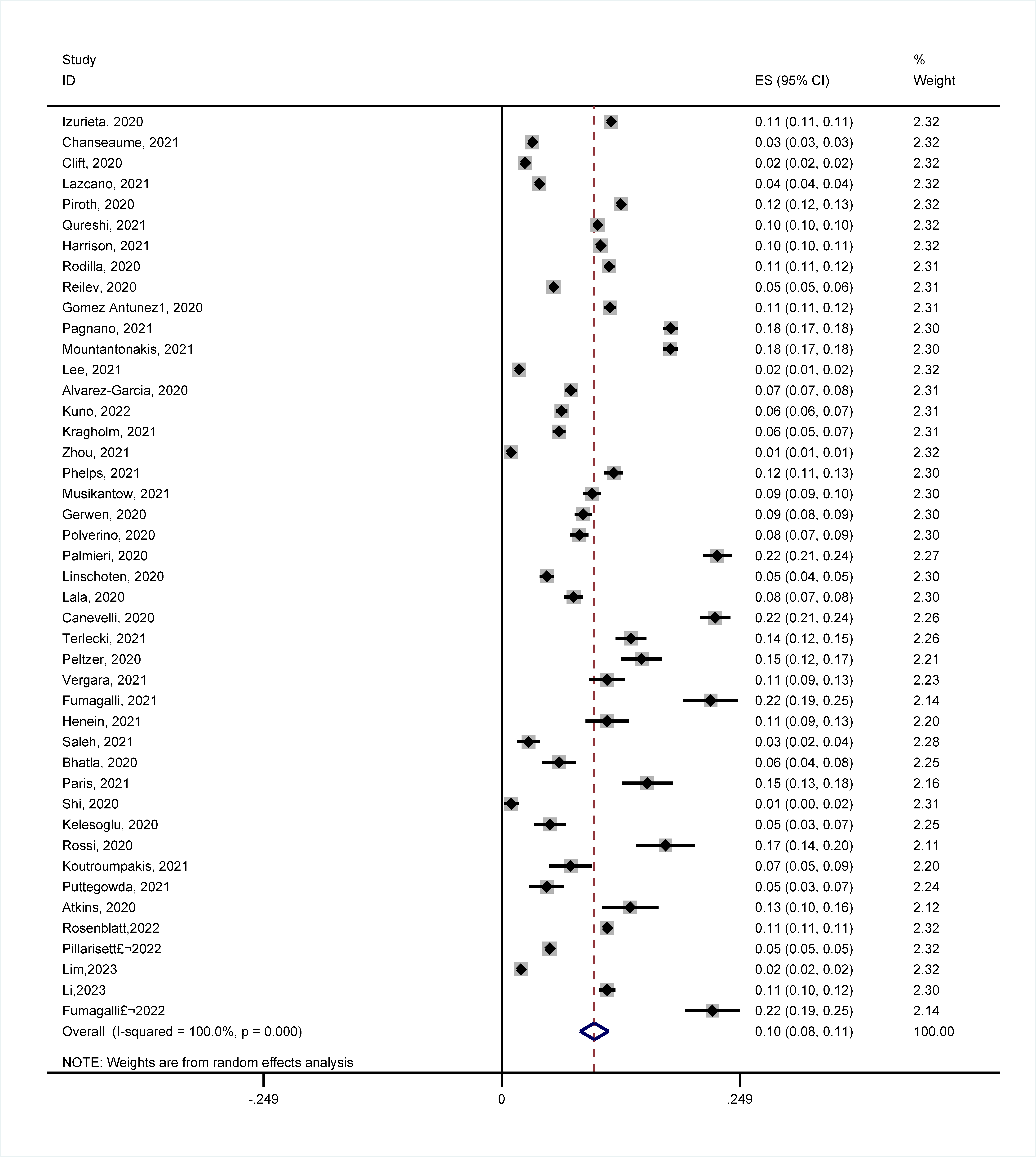
**

**Figure S10. Pooled prevalence of pre-existing rate of AF in sample size > 500**

**
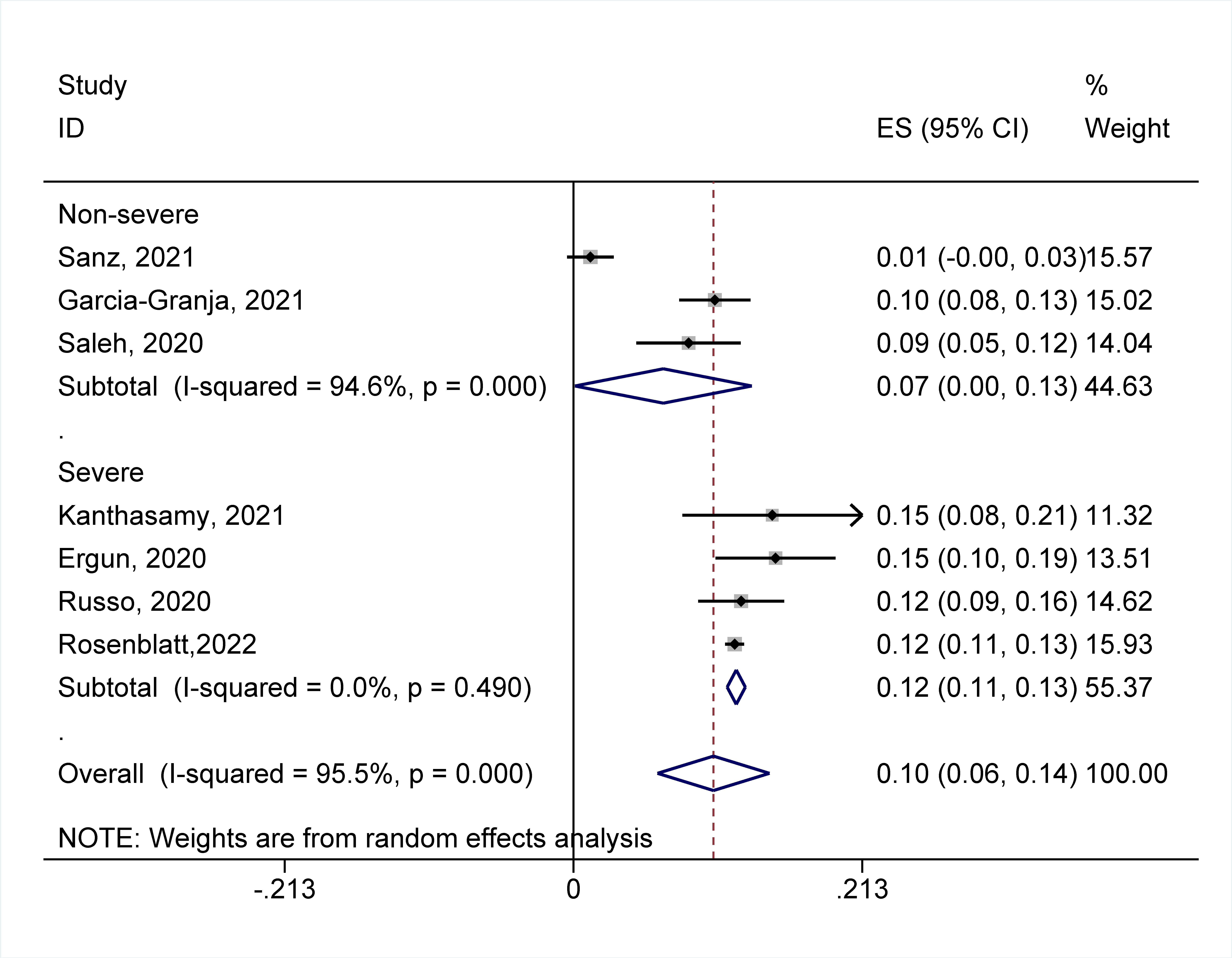
**

**Figure S11. Pooled prevalence of new-onset rate of AF in severe and non-severe patients**

**
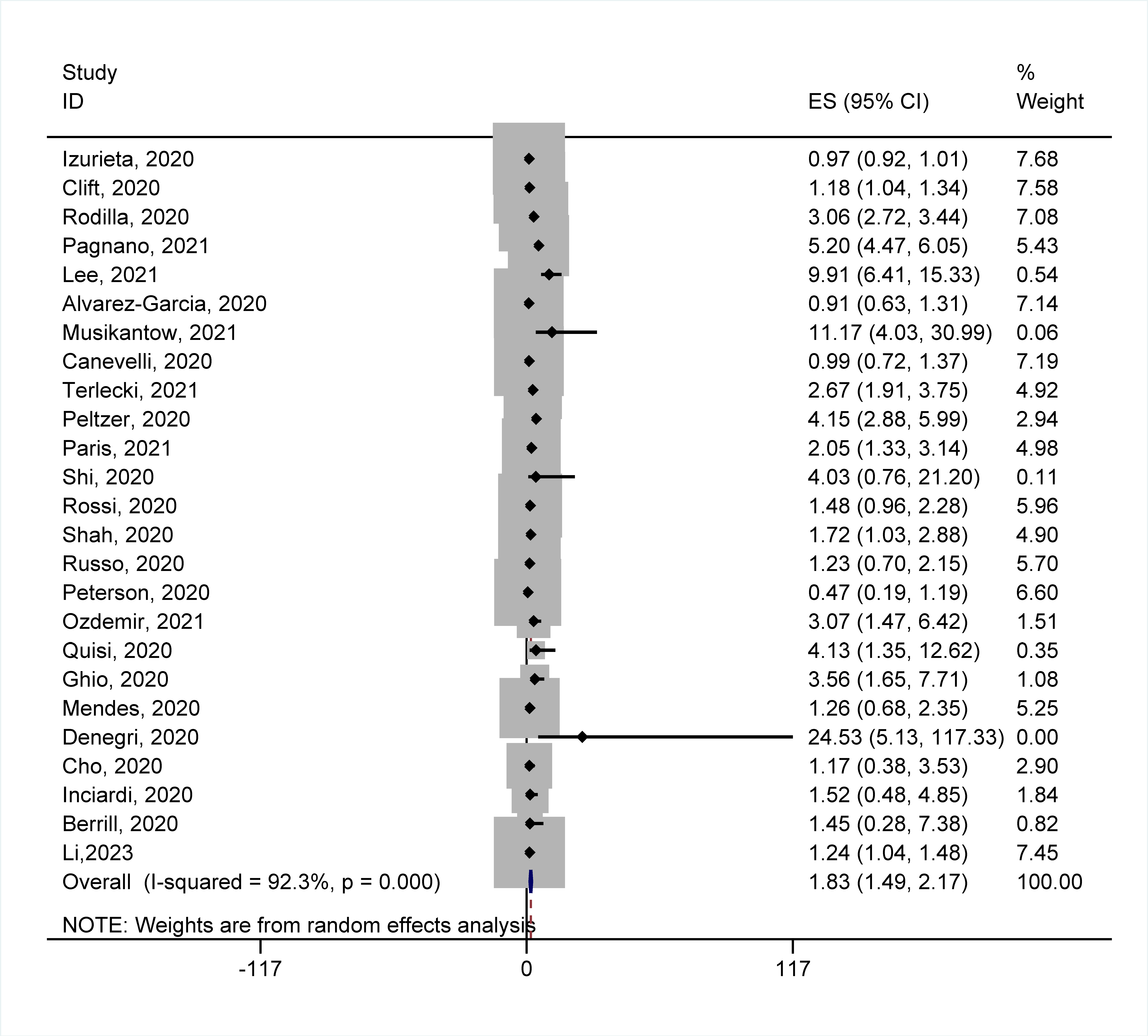
**

**Figure S12. Pooled prevalence of pre-existing AF on all-cause mortality**

**
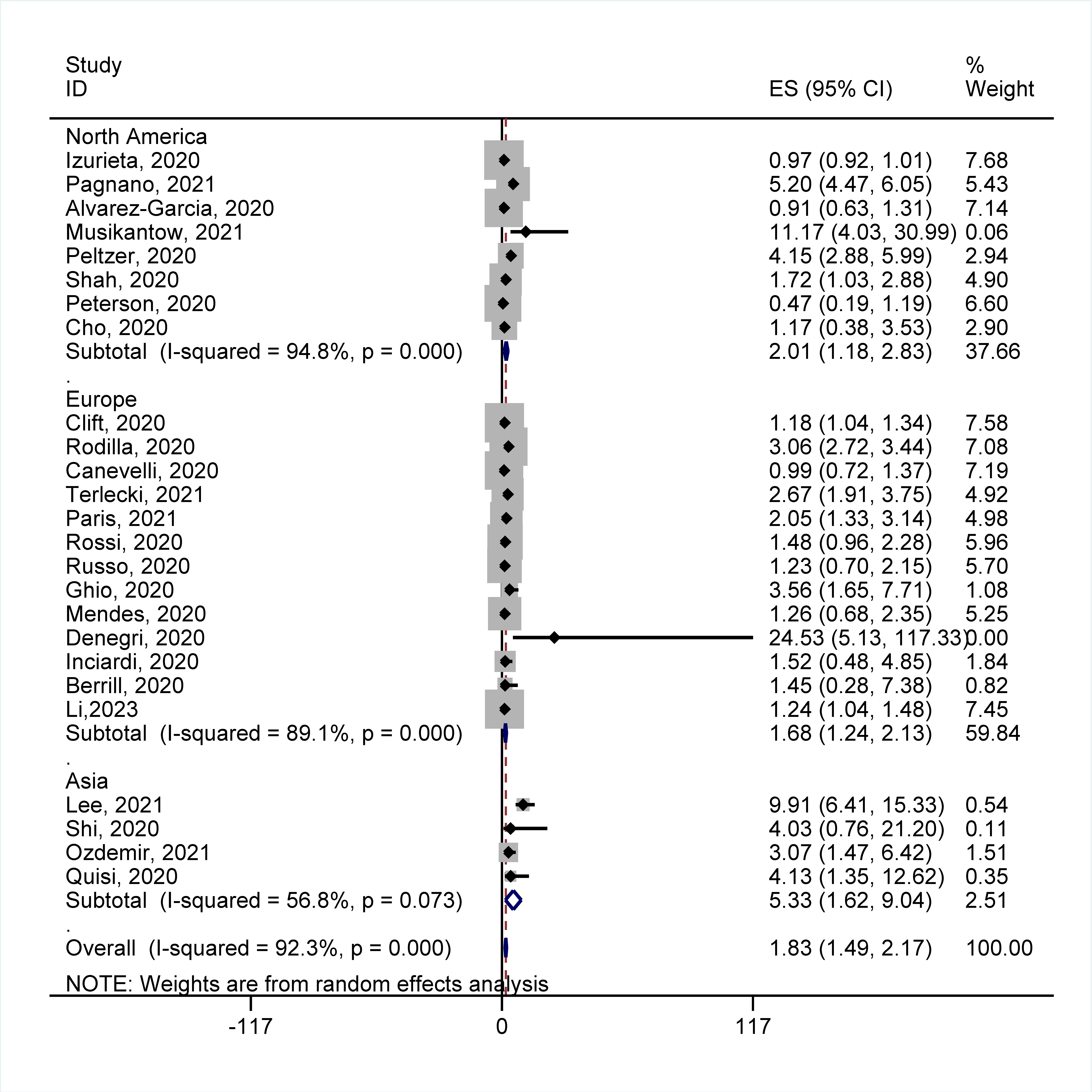
**

**Figure S13. Pooled prevalence of pre-existing AF on all-cause mortality by region**

**
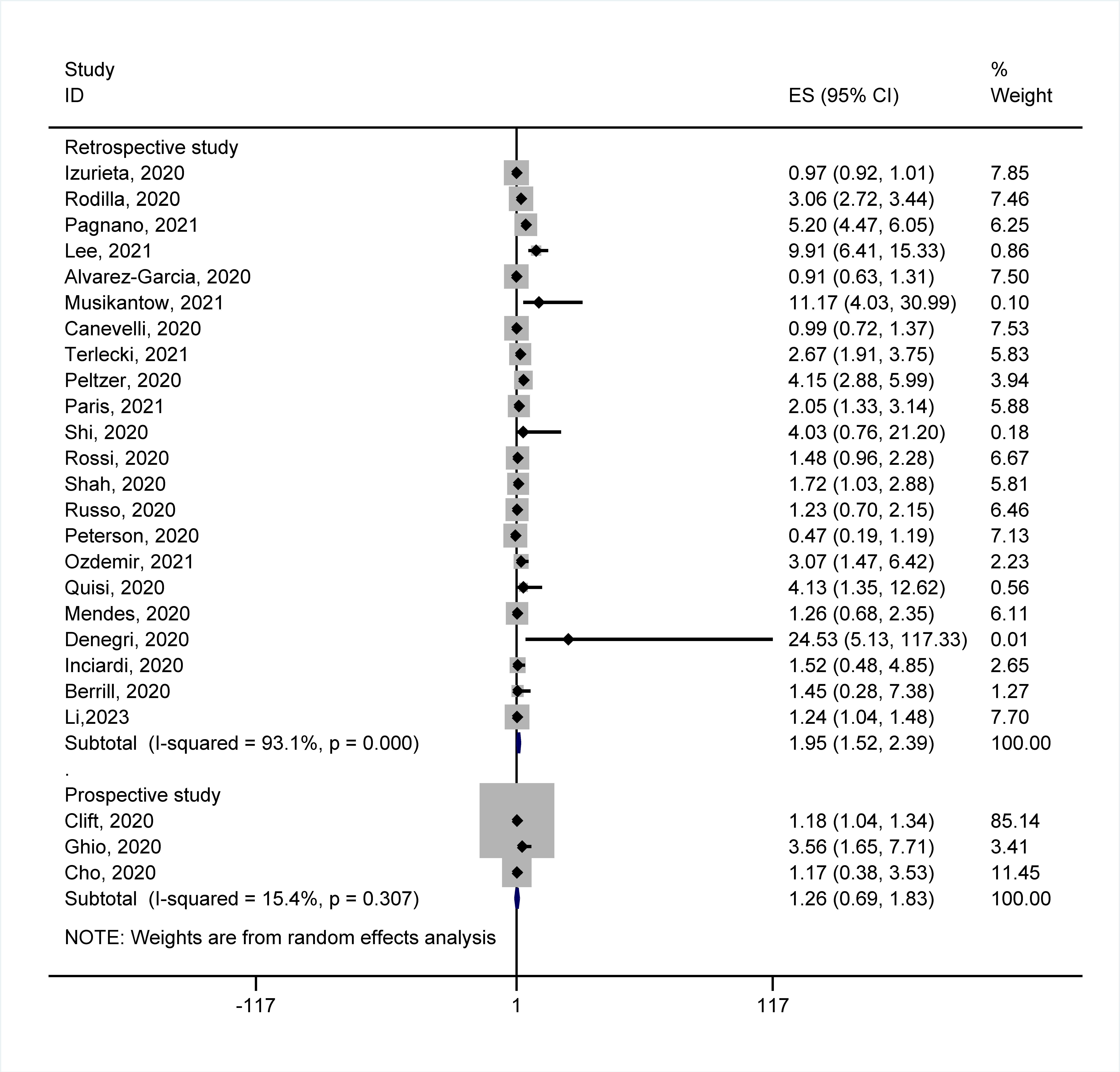
**

**Figure S14. Pooled prevalence of pre-existing AF on all-cause mortality by study type**

**
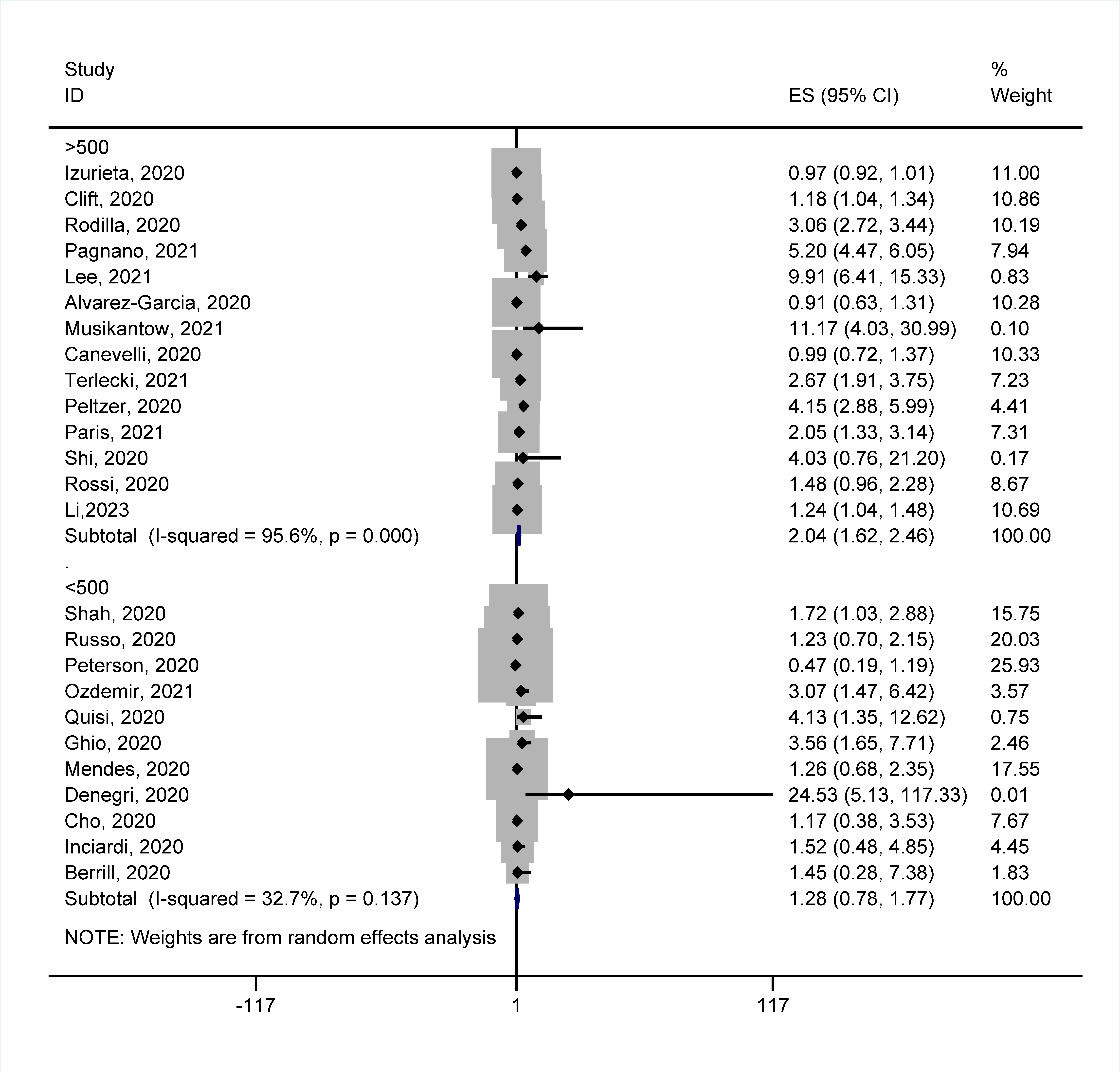
**

**Figure S15. Pooled prevalence of pre-existing AF on all-cause mortality by sample size**

**
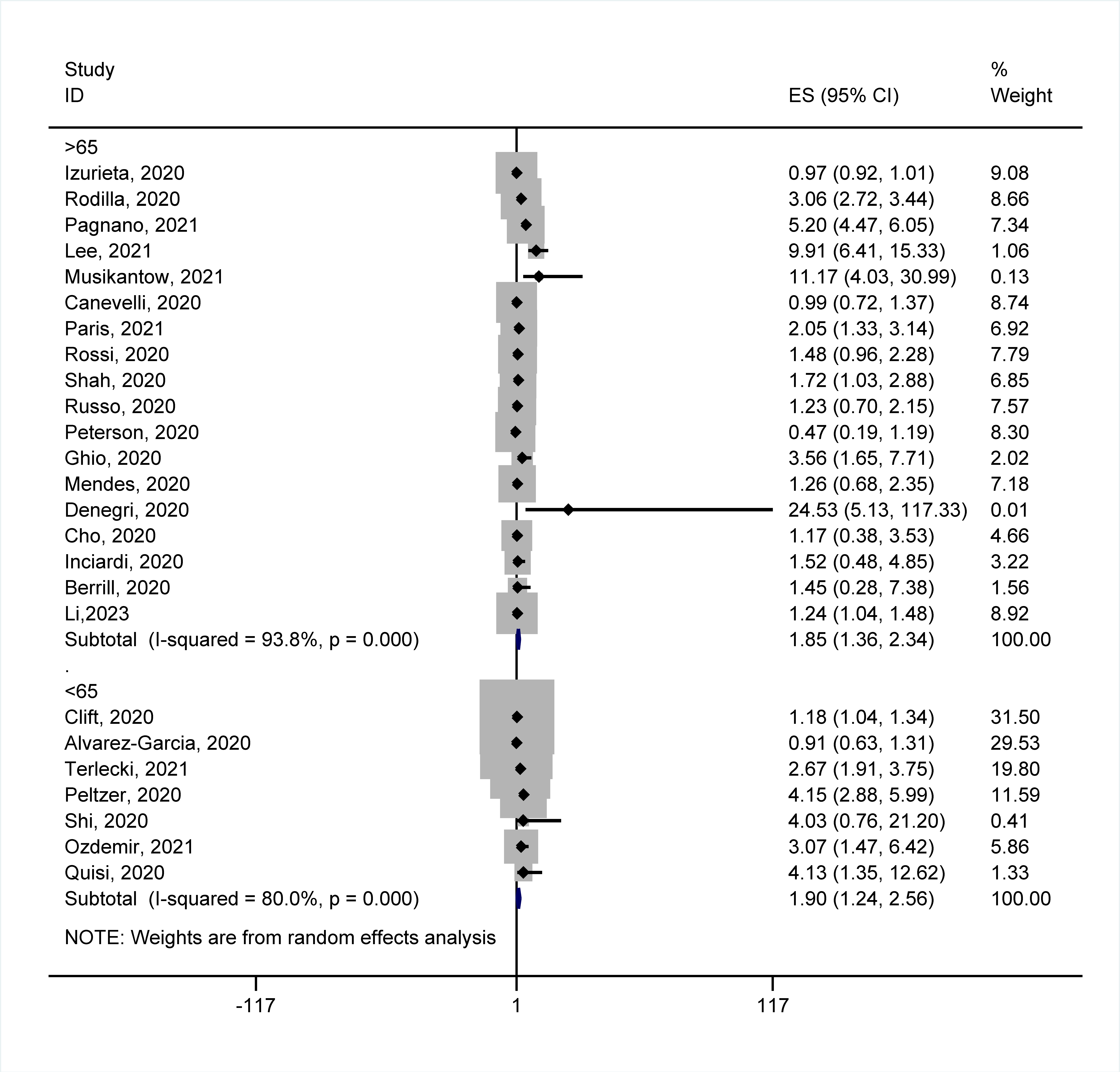
**

**Figure S16. Pooled prevalence of pre-existing AF on all-cause mortality by age**

**
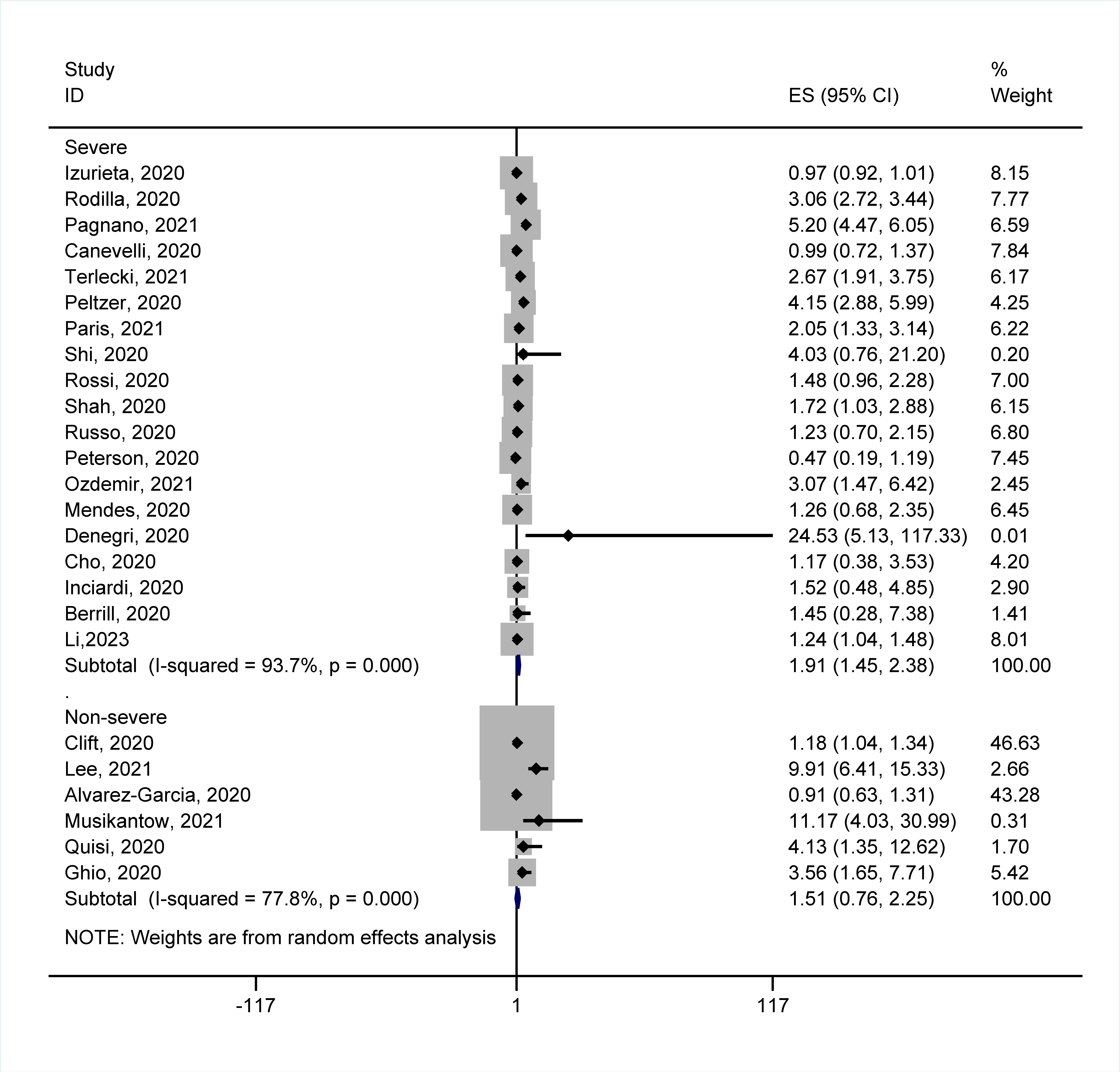
**

**Figure S17. Pooled prevalence of pre-existing AF on all-cause mortality by disease level**

**
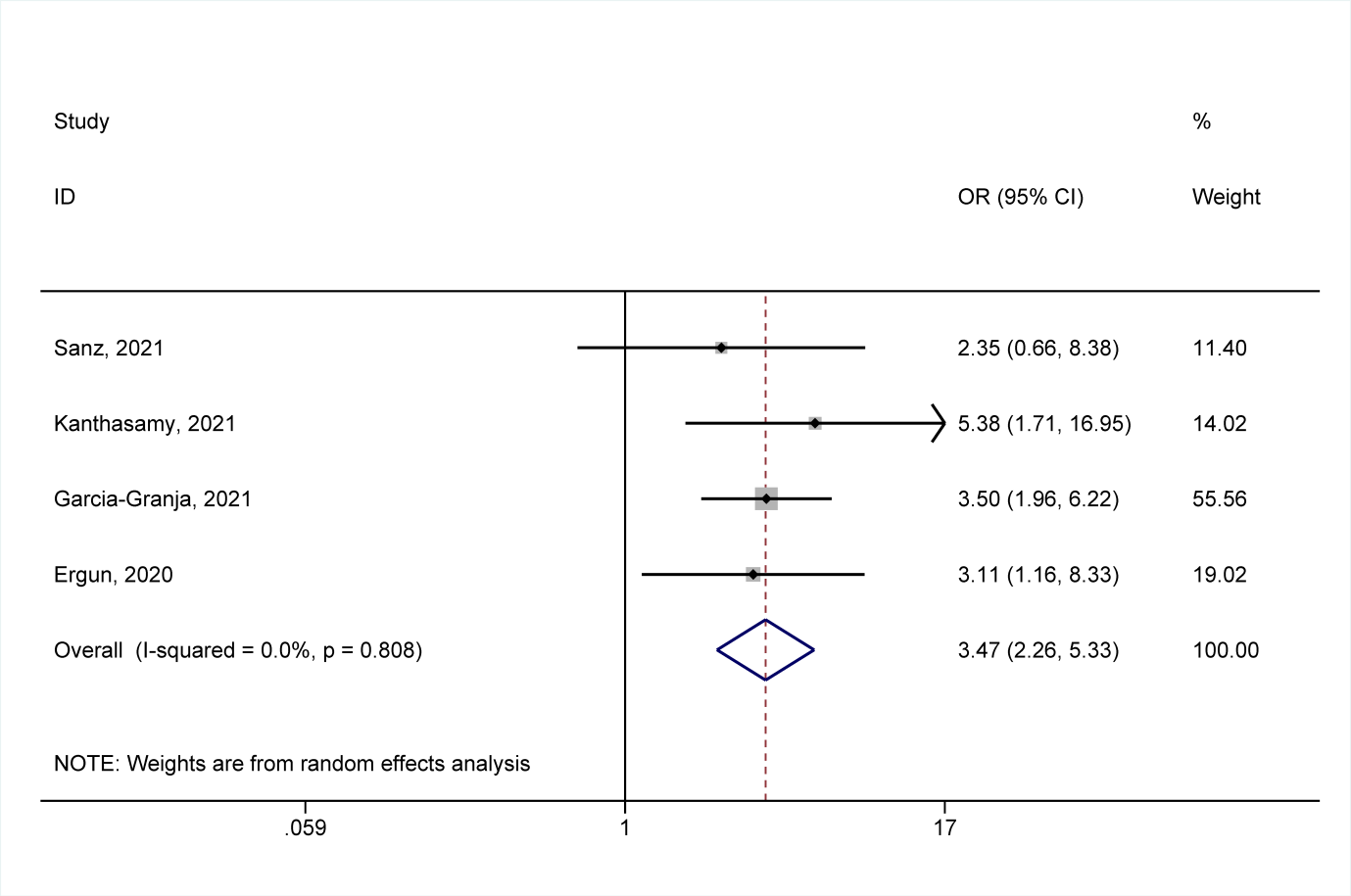
**

**Figure S18. Pooled prevalence of new-onset AF on all-cause mortality**

**
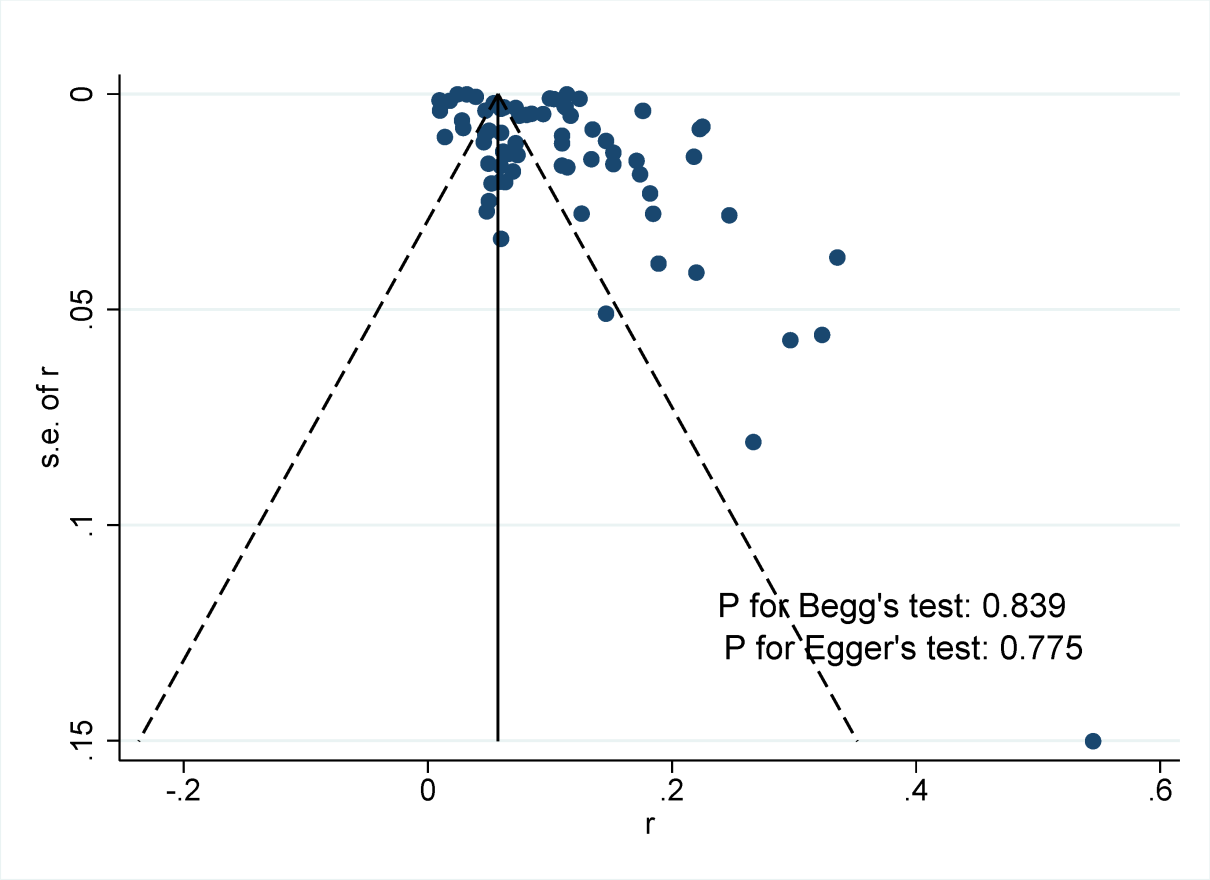
**

# Figure19. Publication bias of studies on the AF prevalence in COVID-patients


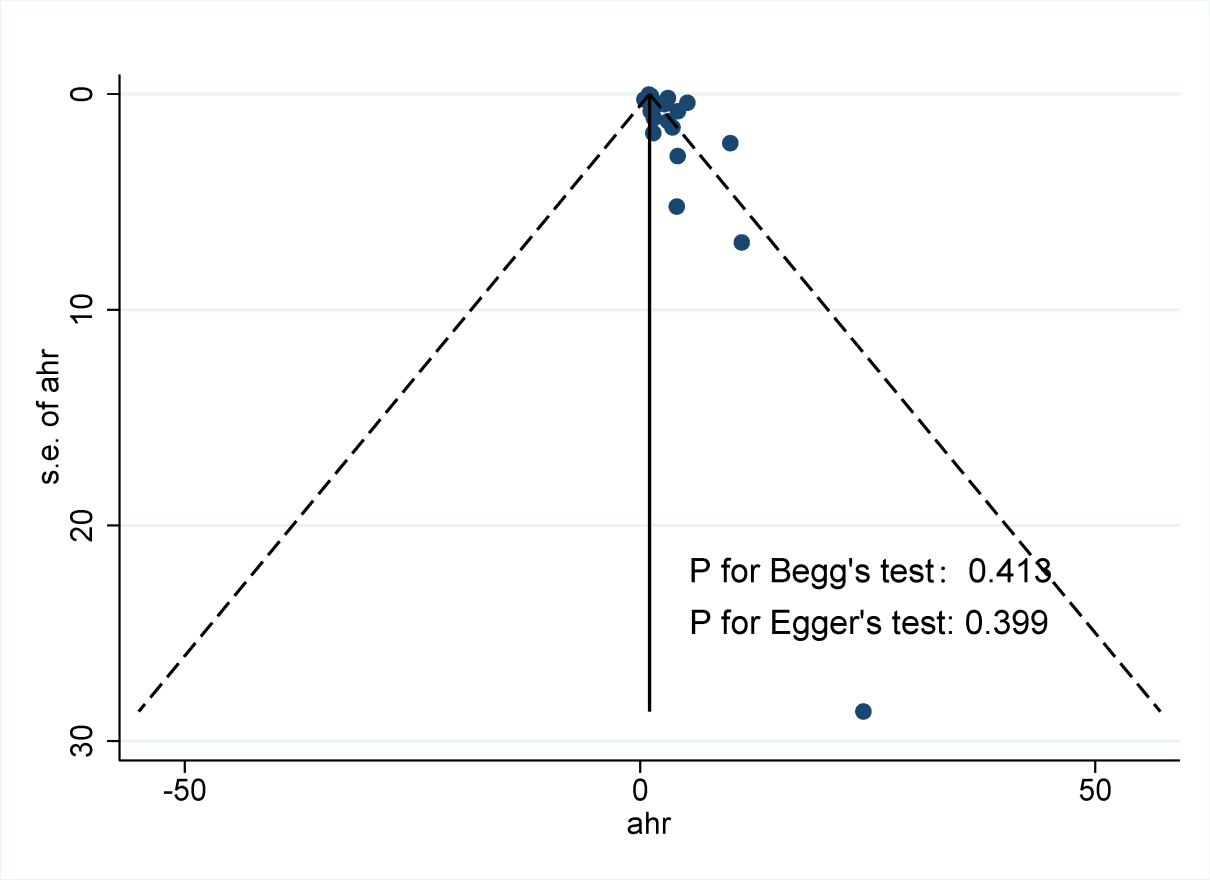


# Figure20. Publication bias of studies on the effect of AF on mortality in COVID-patients

# References

1. Yenerçağ M, Arslan U, Şeker OO, Dereli S, Kaya A, Doğduş M, Öztürk Ç, Akpınar Ç, Şen A. Evaluation of p-wave dispersion in patients with newly diagnosed coronavirus disease 2019. *Journal of cardiovascular medicine (Hagerstown, Md.)*. 2021;22:197-203

2. Wenzler E, Engineer MH, Yaqoob M, Benken ST. Safety and efficacy of apixaban for therapeutic anticoagulation in critically ill icu patients with severe covid-19 respiratory disease. *TH open : companion journal to thrombosis and haemostasis*. 2020;4:E376-E382

3. Wang YJ, Jin QQ, Zheng C, Lin JX, Lin YF, Xu Q, Li J, Lin JF. One-year recording of cardiac arrhythmias in a non-infected population with cardiac implantable devices during the covid-19 pandemic. *International journal of general medicine*. 2021;14:7337-7348

4. Wang Y, Chen L, Wang J, He X, Huang F, Chen J, Yang X. Electrocardiogram analysis of patients with different types of covid-19. *Annals of Noninvasive Electrocardiology*. 2020;25

5. Wallentin L, Lindbäck J, Eriksson N, Hijazi Z, Eikelboom JW, Ezekowitz MD, Granger CB, Lopes RD, Yusuf S, Oldgren J, et al. Angiotensin-converting enzyme 2 (ace2) levels in relation to risk factors for covid-19 in two large cohorts of patients with atrial fibrillation. *European heart journal*. 2020;41:4037‐4046

6. Vila-Córcoles Á, Ochoa-Gondar O, Torrente-Fraga C, Vila-Rovira Á, Satué-Gracia E, Hospital-Guardiola I, de Diego-Cabanes C, Gómez-Bertomeu F, Basora-Gallisà J. [evaluation of incidence and risk profile for suffering covid-19 infection by underlying conditions among middle-aged and older adults in tarragona.]. *Journal of community hospital internal medicine perspectives*. 2020;94

7. Uribarri A, Núñez-Gil IJ, Aparisi Á, Arroyo-Espliguero R, Maroun Eid C, Romero R, Becerra-Muñoz VM, Feltes G, Molina M, García-Aguado M, Cerrato E, Capel-Astrua T, Alfonso-Rodríguez E, Castro-Mejía AF, Raposeiras-Roubín S, Espejo C, Pérez-Solé N, Bardají A, Marín F, Fabregat-Andrés Ó, D'Ascenzo F, Santoro F, Akin I, Estrada V, Fernández-Ortiz A, Macaya C. Atrial fibrillation in patients with covid-19. Usefulness of the cha2ds2-vasc score: An analysis of the international hope covid-19 registry. *Revista espanola de cardiologia*. 2021;74:608-615

8. Tiwari A, Berekashvili K, Vulkanov V, Agarwal S, Khaneja A, Turkel-Parella D, Liff J, Farkas J, Nandakumar T, Zhou T, Frontera J, Kahn DE, Kim S, Humbert KA, Sanger MD, Yaghi S, Lord A, Arcot K, Dmytriw AA. Etiologic subtypes of ischemic stroke in sars-cov-2 patients in a cohort of new york city hospitals. *Frontiers in Neurology*. 2020;11

9. Szarpak L, Filipiak KJ, Skwarek A, Pruc M, Rahnama M, Denegri A, Jachowicz M, Dawidowska M, Gasecka A, Jaguszewski MJ, Iskrzycki L, Rafique Z. Outcomes and mortality associated with atrial arrhythmias among patients hospitalized with covid-19: A systematic review and meta-analysis. *Cardiology journal*. 2021

10. Sotiriou S, Samara AA, Vamvakopoulou D, Vamvakopoulos KO, Sidiropoulos A, Vamvakopoulos N, Janho MB, Gourgoulianis KI, Boutlas S. Susceptibility of β-thalassemia heterozygotes to covid-19. *Journal of clinical medicine*. 2021;10

11. Sala S, Peretto G. Low prevalence of arrhythmias in clinically stable covid-19 patients. *Internal and emergency medicine*. 2020;43:891-893

12. Sabatino J, Ferrero P, Chessa M, Bianco F, Ciliberti P, Secinaro A, Oreto L, Avesani M, Bucciarelli V, Calcaterra G, Calabrò MP, Russo MG, Bassareo PP, Guccione P, Indolfi C, Di Salvo G. Covid-19 and congenital heart disease: Results from a nationwide survey. *Journal of clinical medicine*. 2020;9:1-8

13. Rivera-Caravaca JM, Núñez-Gil IJ, Vivas D, Viana-Llamas MC, Uribarri A, Becerra-Muñoz VM, Trabattoni D, Fernández Rozas I, Feltes G, López-Pais J, El-Battrawy I, Macaya C, Fernandez-Ortiz A, Estrada V, Marín F. Clinical profile and prognosis in patients on oral anticoagulation before admission for covid-19. *European journal of clinical investigation*. 2021;51

14. Peltzer B, Manocha KK, Ying X, Kirzner J, Ip JE, Thomas G, Liu CF, Markowitz SM, Lerman BB, Safford MM, Goyal P, Cheung JW. Outcomes and mortality associated with atrial arrhythmias among patients hospitalized with covid-19. *Journal of cardiovascular electrophysiology*. 2020;31:3077-3085

15. Patel SR, Mukkera SR, Tucker L, Vatsis C, Poma A, Ammar A, Vo M, Khan R, Carlan S. Characteristics, comorbidities, complications, and outcomes among 802 patients with severe acute respiratory syndrome coronavirus 2 in a community hospital in florida. *Critical care explorations*. 2021;3:e0416

16. O'Shea CJ, Middeldorp ME, Thomas G, Harper C, Elliott AD, Ray N, Campbell K, Lau DH, Sanders P. Atrial fibrillation burden during the coronavirus disease 2019 pandemic. *Europace : European pacing, arrhythmias, and cardiac electrophysiology : journal of the working groups on cardiac pacing, arrhythmias, and cardiac cellular electrophysiology of the European Society of Cardiology*. 2021;23:1493-1501

17. Nanjo A, Evans H, Direk K, Hayward AC, Story A, Banerjee A. Prevalence, incidence, and outcomes across cardiovascular diseases in homeless individuals using national linked electronic health records. *European heart journal*. 2020;41:4011-4020

18. Musikantow DR, Turagam MK, Sartori S, Chu E, Kawamura I, Shivamurthy P, Bokhari M, Oates C, Zhang C, Pumill C, Malick W, Hashemi H, Ruiz-Maya T, Hadley MB, Gandhi J, Sperling D, Whang W, Koruth JS, Langan MN, Sofi A, Gomes A, Harcum S, Cammack S, Ellsworth B, Dukkipati SR, Bassily-Marcus A, Kohli-Seth R, Goldman ME, Halperin JL, Fuster V, Reddy VY. Atrial fibrillation in patients hospitalized with covid-19: Incidence, predictors, outcomes, and comparison to influenza. *JACC: Clinical Electrophysiology*. 2021;7:1120-1130

19. Molina I, Marcolino MS. Chagas disease and sars-cov-2 coinfection does not lead to worse in-hospital outcomes. 2021;11:20289

20. Mizuno Y, Hamaki T, Ha ACT, Verma S, Mazer CD, Quan A, Yanagawa B, Latter DA, Yau TM, Jacques F, Brown CD, Singal RK, Yamashita MH, Saha T, Teoh KH, Lam BK, Deyell MW, Wilson M, Hibino M, Cheung CC, Kosmopoulos A, Garg V, Brodutch S, Teoh H, Zuo F, Thorpe KE, Jüni P, Bhatt DL, Verma A. Effect of continuous electrocardiogram monitoring on detection of undiagnosed atrial fibrillation after hospitalization for cardiac surgery: A randomized clinical trial. *Vaccines*. 2021;4:e2121867

21. Lip GYH, Genaidy A, Tran G, Marroquin P, Estes C. Incident atrial fibrillation and its risk prediction in patients developing covid-19: A machine learning based algorithm approach. *European journal of internal medicine*. 2021;91:53-58

22. Holt A, Gislason GH, Schou M, Zareini B, Biering-Sørensen T, Phelps M, Kragholm K, Andersson C, Fosbøl EL, Hansen ML, Gerds TA, Køber L, Torp-Pedersen C, Lamberts M. New-onset atrial fibrillation: Incidence, characteristics, and related events following a national covid-19 lockdown of 5.6 million people. *Journal of arrhythmia*. 2020;41:3072-3079

23. Hedner J, Bertini M, Ferrari R, Guardigli G, Malagù M, Vitali F, Zucchetti O, D'Aniello E, Volta CA, Cimaglia P, Piovaccari G, Corzani A, Galvani M, Ortolani P, Rubboli A, Tortorici G, Casella G, Sassone B, Navazio A, Rossi L, Aschieri D, Rapezzi C. Electrocardiographic features of 431 consecutive, critically ill covid-19 patients: An insight into the mechanisms of cardiac involvement. *The European respiratory journal*. 2020;22:1848-1854

24. Genovesi S, Rebora P. Atrial fibrillation and clinical outcomes in a cohort of hospitalized patients with sars-cov-2 infection and chronic kidney disease. 2021;10

25. Gao P, Wu W, Tian R, Yan X, Qian H, Guo F, Li T, Liu Z, Wang J, Zhou X, Qin Y, Zhao D, Bian X, Lin X, Zhang S. Association between tachyarrhythmia and mortality in a cohort of critically ill patients with coronavirus disease 2019 (covid-19). *Annals of translational medicine*. 2021;9

26. Fumagalli S. Atrial fibrillation and covid-19 in older patients: How disability contributes to shape the risk profile. An analysis of the gerocovid registry. *Journal of interventional cardiac electrophysiology : an international journal of arrhythmias and pacing*. 2021:1-8

27. Denas G, Gennaro N, Ferroni E, Fedeli U, Lorenzoni G, Gregori D, Iliceto S, Pengo V. Reduction in all-cause mortality in covid-19 patients on chronic oral anticoagulation: A population-based propensity score matched study. *International Journal of Cardiology*. 2021;329:266-269

28. Chaudhary R, Padrnos L, Wysokinska E, Pruthi R, Misra S, Sridharan M, Wysokinski W, McBane RD, Houghton DE. Macrovascular thrombotic events in a mayo clinic enterprise-wide sample of hospitalized covid-19–positive compared with covid-19–negative patients. *Mayo Clinic proceedings*. 2021;96:1718-1726

29. Changal K, Mack S. In-hospital outcomes of covid-19 infection in patients with underlying cardiovascular disease. 2021;19:871-876

30. Butt JH, Fosbøl EL, Gerds TA, Andersson C, Kragholm K, Biering-Sørensen T, Andersen J, Phelps M, Andersen MP, Gislason G, Torp-Pedersen C, Køber L, Schou M. All-cause mortality and location of death in patients with established cardiovascular disease before, during, and after the covid-19 lockdown: A danish nationwide cohort study. *European heart journal*. 2021;42:1516-1523

31. Buckley BJR, Harrison SL, Fazio-Eynullayeva E, Underhill P, Lane DA, Lip GYH. Prevalence and clinical outcomes of myocarditis and pericarditis in 718,365 covid-19 patients. *European journal of clinical investigation*. 2021;51:e13679

32. Brojakowska A, Eskandari A, Bisserier M. Comorbidities, sequelae, blood biomarkers and their associated clinical outcomes in the mount sinai health system covid-19 patients. 2021;16:e0253660

33. Boytsov SA, Pogosova NV, Paleev FN, Ezhov MV, Komarov AL, Pevsner DV, Gruzdev KA, Barinova IV, Suvorov AY, Alekseeva IA, Milko OV. Clinical characteristics and factors associated with poor outcomes in hospitalized patients with novel coronavirus infection covid-19. *European heart journal. Case reports*. 2021;61:4-14

34. Bhatia KS, Sritharan HP, Chia J, Ciofani J, Nour D, Chui K, Vasanthakumar S, Jayadeva P, Kandadai D, Allahwala U, Bhagwandeen R, Brieger DB, Choong CYP, Delaney A, Dwivedi G, Harris B, Hillis G, Hudson B, Javorsky G, Jepson N, Kanagaratnam L, Kotsiou G, Lee A, Lo STH, MacIsaac AI, McQuillan BM, Ranasinghe I, Walton A, Weaver J, Wilson W, Yong A, Zhu J, van Gaal W, Kritharides L, Chow C, Bhindi R. Cardiac complications in patients hospitalised with covid-19 in australia. *Heart, lung & circulation*. 2021;30:1834-1840

35. Barbhaiya CR, Wadhwani L, Manmadhan A, Selim A, Knotts RJ, Kushnir A, Spinelli M, Jankelson L, Bernstein S, Park D, Holmes D, Aizer A, Chinitz LA. Rebooting atrial fibrillation ablation in the covid-19 pandemic. *Journal of Interventional Cardiac Electrophysiology*. 2021

36. Abrams MP. Clinical and cardiac characteristics of covid-19 mortalities in a diverse new york city cohort. *Revista da Associacao Medica Brasileira (1992)*. 2020;31:3086-3096
